# The BACHD Rat Model of Huntington Disease Shows Signs of Fronto-Striatal Dysfunction in Two Operant Conditioning Tests of Short-Term Memory

**DOI:** 10.1371/journal.pone.0169051

**Published:** 2017-01-03

**Authors:** Erik Karl Håkan Clemensson, Laura Emily Clemensson, Olaf Riess, Huu Phuc Nguyen

**Affiliations:** 1 Institute of Medical Genetics and Applied Genomics, Tuebingen, Germany; 2 Centre for Rare Diseases, Tuebingen, Germany; 3 QPS Austria, Grambach, Austria; Emory University, UNITED STATES

## Abstract

The BACHD rat is a recently developed transgenic animal model of Huntington disease, a progressive neurodegenerative disorder characterized by extensive loss of striatal neurons. Cognitive impairments are common among patients, and characterization of similar deficits in animal models of the disease is therefore of interest. The present study assessed the BACHD rats' performance in the delayed alternation and the delayed non-matching to position test, two Skinner box-based tests of short-term memory function. The transgenic rats showed impaired performance in both tests, indicating general problems with handling basic aspects of the tests, while short-term memory appeared to be intact. Similar phenotypes have been found in rats with fronto-striatal lesions, suggesting that Huntington disease-related neuropathology might be present in the BACHD rats. Further analyses indicated that the performance deficit in the delayed alternation test might be due to impaired inhibitory control, which has also been implicated in Huntington disease patients. The study ultimately suggests that the BACHD rats might suffer from neuropathology and cognitive impairments reminiscent of those of Huntington disease patients.

## Introduction

Huntington disease (HD) is an autosomal hereditary neurodegenerative disease, which is caused by a specific mutation in the gene for the huntingtin protein [1,2]. The gene contains a CAG repeat sequence in its first exon, which codes for a stretch of glutamines that is present in the translated protein. Patients who carry an allele with a CAG repeat sequence that is 40 repeats or longer invariably develop HD. As the disease manifests and progresses there is extensive neuronal loss throughout the brain. This is first evident in the caudate nucleus of the striatum, although it eventually affects most brain regions. This result in a wide range of clinical signs that are commonly grouped into motor, psychiatric, cognitive and metabolic symptoms. There are currently no disease-modifying treatments available for HD, and the disease is invariably fatal.

HD patients have been found to suffer from a range of different cognitive impairments [3–14]. Among these there are frequent findings indicating impaired executive function [10–14], which is commonly considered to be dependent on specific regions of the prefrontal cortex and their connections to various subcortical nuclei [15–18]. In line with this, some of the executive function impairments seen in HD appear to be related to fronto-striatal pathology [19–22]. Due to the single disease-causing gene of HD, there are several relevant transgenic animal models of the disease. Our group works primarily with the BACHD rat, a recently developed model that is currently being characterized in order to understand its advantages and disadvantages concerning modeling of HD. In the current study, we investigated the rats’ performance in two operant conditioning protocols called the delayed alternation and the delayed non-matching to position tests. Both are frequently used for assessing short-term memory in rodents, and commonly utilize operant conditioning chambers equipped with two retractable levers [23–29]. In the alternation test, the rats have to learn to alternate their responses between the two levers, when these are presented on discrete trials. In the non-matching test, trials are divided into two parts. During the first part, the rats are presented with one randomly chosen sample lever. During the second part, the rats are presented with both levers and should respond to the lever that was not presented as a sample. Successful performance in either protocol is rewarded with small food pellets. In order to evaluate the rats’ short-term memory, delays are introduced in the protocols to evaluate how long the rats remember which lever to respond to. As successful performance in both the delayed alternation and the delayed non-matching to position test is sensitive to various lesions of prefrontal and striatal brain regions [23–29], they offer a good set of tests to evaluate the presence of HD-related pathology in the BACHD rats.

## Materials and Methods

### Animals

A total of 48 male rats were used for the study. These were acquired from two separate in-house breeding events with hemizygous BACHD males from the TG5 line [30] paired with WT females (Charles River, Germany). All animals were on Sprague-Dawley background. Animals were genotyped according to previously published protocols [30] and housed in genotype-matched groups of three in type IV cages (38×55 cm), with high lids (24.5 cm from cage floor). During tests, rats were food restricted according to the two protocols described below. During both protocols, each cage was given a specific daily amount of food (SNIFF V1534-000 standard chow) to maintain appropriate restriction levels. Rats had free access to food between the tests. Rats had free access to water through the entire study. During tests, body weight was measured daily to track the rats’ relative food restriction level and assess basic health. Between tests, body weight was measured weekly.

The animal facility kept 21–23°C, 55–10% humidity, and was set to a partially inverted light/dark cycle with lights on/off at 02:00/14:00 during summer, and 01:00/13:00 during winter.

Two groups of 24 rats were formed from the total of 48. The birth dates of the rats in these two groups were spaced roughly two months apart. Each group was composed of 12 WT and 12 BACHD rats. One group was used for a longitudinal study of performance on the delayed alternation protocol, while the other one was used for a longitudinal study of performance on the delayed non-matching to position protocol. The groups were run in an alternating fashion so that the testing ages were the same for both groups. Behavioral evaluation was thus performed at 4, 9, 14 and 19 months of age. It should, however, be noted that training was initiated approximately two months before the set ages, as the rats had to progress through several steps before reaching the final test protocols. Thus, the actual test ages were 2–4, 7–9, 12–14 and 17–19 months of age.

During the late test ages, several rats had to be sacrificed due to illnesses (the exact number of rats is specified in the Results section). Decision to sacrifice was always made together with the local veterinarians, after careful examination of the rat. End points considered unidentified illnesses causing weight loss past 80% of free-feeding body weight, or critically reduced welfare according to commonly used indicators (i.e. tumorous swellings that clearly impaired the rats’ ability to eat, move and clean themselves, labored breathing, poor grooming, lethargy, disturbed gait, sensitivity to handling or reduced appetite). In such cases, these rats were euthanized in a CO_2_ inhalation chamber. No other methods were used to alleviate suffering.

All tests were approved by the local ethics committee (Regierungspraesidium Tuebingen) and carried out in accordance with the German Animal Welfare Act and the guidelines of the Federation of European Laboratory Animal Science Associations, based on European Union legislation (Directive 2010/63/EU).

### Food restriction protocols

Two different food restriction protocols were used during the study. The first one focused on restricting the animals to a specific relative body weight. During this, both BACHD and WT rats were restricted until they reached 85% of their respective free-feeding body weight. This relative body weight, or food restriction level, was calculated using previously gathered data from growth curves of free-feeding BACHD and WT rats. Thus, the calculations of restriction levels were made with gender, age and genotype-matched values and took normal growth into account. This protocol was used as the start point at all test ages and will be referred to as the standard food restriction protocol.

We have previously found that male BACHD rats are obese, but have comparable body weights to WT rats [31]. Interestingly, the transgenic rats still reliably consume less food than their WT littermates [30,31]. It is currently unclear to what extent these phenotypes affect the BACHD rats’ motivation to perform food-oriented tasks in general, although it has been shown that they are less motivated than WT rats to perform a progressive ratio task (a classical test of motivation) when standard food restriction protocols are used [31]. Because of this, we sought to evaluate the impact of motivation on the readouts from the protocols used in the current study. Thus, once data from performance during the standard food restriction protocol had been gathered, the restriction protocol was changed to an alternative protocol. During this, the amount of food given to WT rats was increased so that they reached 95% of their free-feeding weight rather than the previous 85%. When they had reached the new restriction level, data for a second baseline was gathered. BACHD rats were during this given continuous training (but were kept at 85% of their free-feeding body weights) to validate that any effects seen in the WT rats were indeed due to the change in food restriction level.

It should be noted that it was rarely possible to give the exact same amount of food to either of the genotypes during extended periods of time, as both the standard and alternative restriction protocol had to take natural growth into account. We have, however, found that these smaller adjustments have little impact on the rats’ performance.

### Operant conditioning setup

A bank of six operant conditioning boxes (Coulbourn Instruments, H10-11R-TC) was used to run the test. Each chamber was equipped with two retractable levers, one on either side of a central pellet delivery trough that was equipped with a yellow light. This light was used to signal the delivery of a reward pellet during the protocols. Above each lever was a single white cue light. The boxes further contained a red house light on the wall opposite from the levers and pellet delivery trough, which shone during the full duration of the training sessions. A water bottle was also available on this wall to ensure *ad libitum* access to water during testing. The protocols were designed and run with Graphic State 4.1.04. Rats were given single daily sessions, meaning that a total of four daily runs with all six operant chambers were needed to assess a full group. Each run assessed three WT and three BACHD rats in a determined order so that a given rat was trained on the same time of day through all tests. Each rat was assigned to a specific operant chamber, although this was arranged so that each operant chamber was used to assess equal numbers of WT and BACHD rats.

Behavioral assessment started approximately six hours after dark phase onset, in a room separate from the animals’ housing room, using soft red light. Rats received their daily amount of regular food one hour after the completion of the last run of the day.

### Operant conditioning protocols

At each test age, the rats were first put on food restriction for approximately 14 days before any training occurred. This aimed at restricting both WT and BACHD rats to 85% of their respective free-feeding body weights, as described above. During the first test age, this step was also used to familiarize the rats with the reward pellets that were used in the operant conditioning boxes. This was done by adding a spoonful of reward pellets (Bio-serv, Dustless Precision Pellets^®^ F0021, purchased through Bilaney Consultants, Duesseldorf, Germany) to the daily amount of food given to each cage. It was not necessary to repeat this when the rats were reassessed at older ages.

Before reaching the final operant conditioning protocols of interest, the rats had to be trained in a series of separate protocols. The first protocols aimed at habituating the rats to the operant conditioning boxes, and at training them to reliably respond to the levers. These first steps were similar for the two rat groups and were only run during the first test age. The specific protocols are described below.

#### Habituation

All rats were given two habituation sessions in order for them to familiarize themselves to the operant conditioning boxes and the pellet trough where food rewards could be retrieved. During these sessions, both levers were retracted and a single reward pellet was delivered to the pellet trough at 10-, 15-, 20-, 25-, or 30-second intervals. The pellet delivery interval varied in a pseudo-randomized fashion so that each set of five deliveries used each interval once. Pellet retrieval, or failure to retrieve the pellet within five seconds after delivery, lead to the start of the next pellet delivery interval. Pellet deliveries were signaled by the light in the pellet trough being switched on. The light was switched off when the pellet was retrieved, or when five seconds had passed and the next interval started. Sessions lasted until 100 pellets had been delivered, which took roughly 30 minutes.

#### Continuous reinforcement (CRF) with help

The aim of these sessions was to train the rats to reliably perform lever pushes to obtain reward pellets. During the sessions one of the two levers was inserted into the box and remained inserted until the end of the session. Each lever push resulted in the delivery of a single reward pellet. At the start of the session, the lever was baited with a paste made by mashing some reward pellets in water. The experimenter then manually delivered rewards when the rats approached, sniffed and touched the inserted lever. Through this, the rats eventually performed a few accidental responses and soon developed a reliable lever-pushing behavior. Sessions ended either after 30 minutes had passed or after 100 pellets had been delivered. Training continued until the rats had managed to perform 100 pushes within one session without any help from the experimenter. Training was organized so that half of the rats from each genotype group were trained on the right lever, while the other half was trained on the left lever.

#### CRF on the second lever

Once the rats had passed the criterion for CRF performance on the first lever, the same training was done for the second lever. Thus, the lever the rats were initially trained on was retracted, while the other lever was inserted. The new lever was also baited at the start of the trial, but the experimenter only manually delivered reward pellets if rats had clear problems understanding what to do. Session durations and criteria were the same as during the initial CRF training.

#### Forced alternation and non-matching to position sequence training

At this point, the rats of the two groups were trained on slightly different protocols. Both protocols aimed at training the rats to reliably start the individual trials that made up the delayed alternation and delayed non-matching to position sessions. In addition, the protocols sought to familiarize the rats with the main concept of the tasks they were going to perform (i.e. alternation and non-matching to position).

The rats in the delayed alternation group were trained on a forced alternation protocol. For this protocol, each session was split into a series of trials, separated by brief (2 s) inter-trial intervals (ITIs). The sessions started with an ITI step, with both levers retracted, the house light switched on and all cue lights off. At the end of the ITI the light in the pellet trough would start to shine. When the rats entered the pellet trough with their head, the light was switched off and either the left or right lever was inserted. The lever remained inserted until the rats performed a response. The lever retracted and a reward pellet was delivered at the off signal of a lever response. Delivery of a reward pellet was signaled with the light in the pellet trough shining once again. The trial ended either when the rats collected the reward pellet or when five seconds had passed since the reward pellet had been delivered. Either event triggered the start of a new ITI. On the first trial of the session, the protocol was set to randomly insert either the left or the right lever. On all subsequent trials, the inserted lever would be on the opposite side of the lever used during the previous trial. Through this, the rats were forced to alternate their responses between the left and right lever. The sessions lasted either until the rats had completed 100 trials or until 45 minutes had passed. Rats were trained until they completed 100 trials within the session duration limit without any help from the experimenter.

The rats in the delayed non-matching to position group were trained on a non-matching to position sequence training protocol. The sessions of this protocol were also split into a series of trials separated by ITIs. The duration of these ITIs varied in a pseudo-randomized fashion between 5, 7, 9 and 11 seconds so that each block of four ITIs used each duration once. The sessions started with an ITI step, with both levers retracted, the house light switched on and all cue lights off. At the end of the ITI the light in the pellet trough started to shine. When the rats entered the pellet trough with their head, the light was switched off and either the left or right lever was inserted. The protocol followed a pseudo-randomized structure so that each block of six trials used three trials with the left lever and three trials with the right lever. This also meant that a given trial type (i.e. left or right lever) could maximally appear six times in a row. The lever remained inserted until the rats performed a lever response. The lever retracted and the food trough light started to shine again at the off signal of a lever response. Notably, no reward pellet was given for this response. When the rats entered the pellet trough again, the pellet trough light went out, both lever cue lights shone and the lever on the opposite side from the first one was inserted. The lever once again stayed inserted until the rats made a response. The lever retracted, the lever cue lights stopped shining and a reward pellet was delivered at the off signal of a lever response. As in previous steps, the delivery of a reward pellet was signaled by the light in the pellet trough starting to shine. The trial ended either when the rats collected the reward pellet, or when five seconds had passed since the reward pellet was delivered. Either event triggered the next ITI. The sessions lasted either until rats had completed 100 trials or until 45 minutes had passed. Rats were trained until they completed 100 trials within the session duration limit without any help from the experimenter.

Once rats had reached the performance criterion on their respective protocols, omission limits were added in the protocols to make sure that the rats performed the desired responses at a proper pace. For the forced alternation protocols, these limits were set for starting a trial and responding to the inserted levers. For the non-matching to position sequence learning, the limits were set for the trial start, responding to the first lever, returning to the pellet trough and responding to the second lever. On those steps, if a rat failed to perform the required response within ten seconds, the protocol went into an omission state, in which all lights were switched off and all levers retracted. After ten seconds the protocols went into ITIs that ensured that the rats would be given an identical trial to the one they had just failed to complete. These protocols were run until the rats performed less than 5 omissions in total, while completing 100 trials within the session duration. Importantly, omitted trials were not counted towards the 100-trial limits of the sessions, as they were not considered to be completed trials.

#### Free alternation and non-matching to position

**Fig 1:**
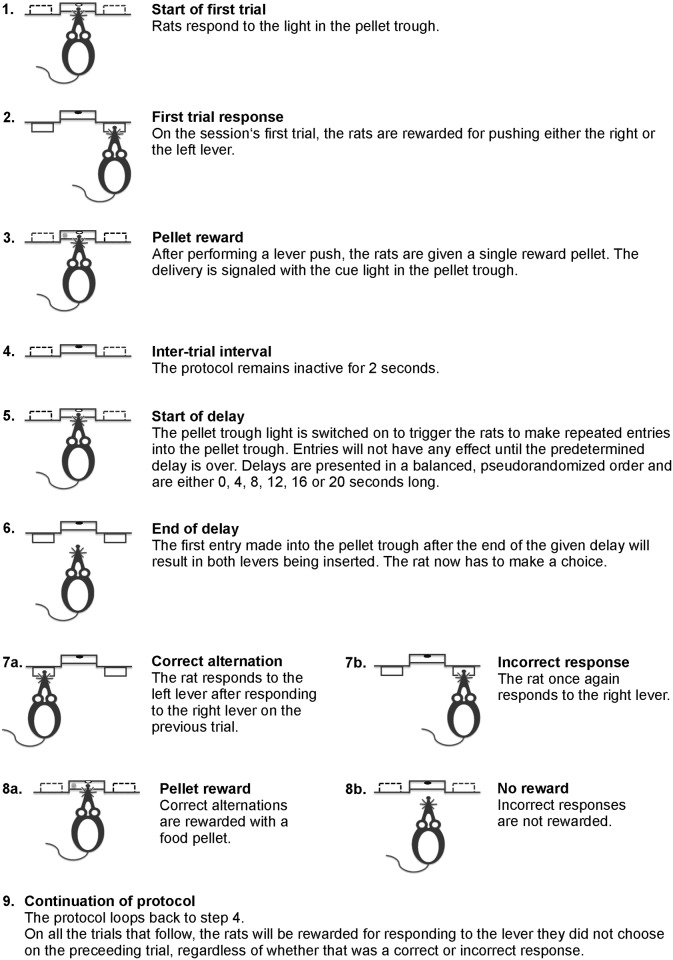
Delayed alternation protocol. The figure describes the steps that make up individual trials in the delayed alternation test.

**Fig 2:**
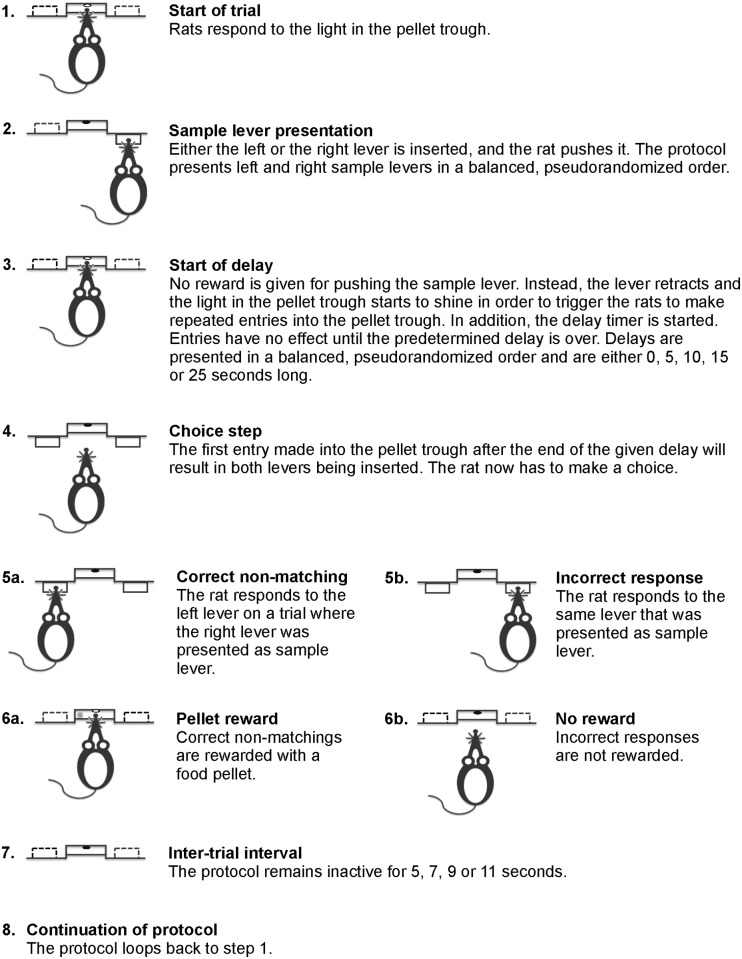
Delayed non-matching to position protocol. The figure describes the steps that make up individual trials in the delayed non-matching to position protocol.

The next set of protocols were the first ones where rats were able to make mistakes, and also served as the starting point when rats were retrained at older ages. The basic structure of the protocols were similar to the forced alternation and the non-matching to position sequence learning protocols. Thus, they used the same basic structure concerning the start and stop of the individual trials as well as the ITI setup described above. In addition, both protocols still ended either after 100 completed trials or 45 minutes. As above, omitted trials did not count towards this 100-trial limit, while both successful and failed trials did. The outlines of the two tests are shown in Figs 1 and 2.

The main difference between the forced and free alternation protocols was that both levers were inserted during each trial of the latter protocol. On the first trial of each session, the rats were rewarded for pushing either the left or right lever. On all subsequent trials, however, the rats were only rewarded for pushing the lever they did not respond to on the previous trial. Importantly, this was independent of whether the previous trial was successful or not. Thus, in order to continuously receive rewards, the rats had to alternate their responses between the left and right levers and avoid making repeated responses on one of the levers. A mistake resulted in a brief timeout (3 s) during which the house light was switched off. At the end of the timeout, the protocol returned to an ITI state, which was followed by a new trial. On occasions where the rats performed an omission, the protocol reset to a starting position. Thus, on the next trial the rats were rewarded for pushing either lever, and this trial set the start point for the next series of alternations. Importantly, the first trial of the session, and trials that directly followed omissions, were not counted towards the 100-trial limit of the session and were also not included in the success rate calculations. Training continued until the rats showed an 85% or higher success rate during three consecutive sessions.

The free non-matching to position test used the same two-part structure as the non-matching to position sequence learning protocol. Thus, after the trial start, the rats were prompted to push a single non-reinforced lever, which will be referred to as the sample lever. After responding to the sample lever and returning to the pellet trough, both levers were now inserted into the box. This second part of the non-matching trials will be referred to as the choice step. Similar to the previous training step, the rats were rewarded for pushing the lever that was not presented during the sample step. Pushing the same lever as the sample lever resulted in a ten-second timeout similar to the one described for the free alternation protocol. During the first testing age, the rats were trained until they showed an 85% success rate or higher during three consecutive sessions. This was, however, reduced to two consecutive sessions when the rats were retested, as the rats showed little problem with handling the test. Trials using the left and right sample lever were pseudo-randomized as described above. Omissions resulted in the rats being presented with a trial using the same sample lever again.

#### Delayed alternation and non-matching to position

When the rats had learned to handle their respective basic task, delays were introduced into the protocols. The aim of this was to assess the rats’ short-term memory function. In the alternation protocol, the delays were introduced at the start of the trials, at the point when the pellet trough light started to shine. As explained above, a head entry at that point usually triggered the continuation of the trial (i.e. lever insertion). During the delayed alternation, however, head entries had no effect until the end of the set delay. Once the delay was over, the first head entry resulted in the levers being inserted. Delays were introduced in a similar manner in the non-matching to position protocol. Specifically, they were used during the step where the rats returned to the pellet trough after responding to the sample lever. As described above, making the second entry into the pellet trough would usually result in triggering the choice step of the protocol. But during the delayed non-matching to position protocol, head entries had no effect until the delay was over. Similarly to the delayed alternation protocol, the first head entry performed after the end of the delay would trigger the choice step. Through these delays, rats were thus forced to perform responses in the two protocols with certain specific spacing in relation to either the previous trial or their sample lever response. The omission limits that were set for trial start and initiation of the choice step were applied at the end of the delays. Thus, if a rat had not performed a head entry response within ten seconds after the end of the delay on either protocol, the trial was aborted.

The sessions were initially made up of 100 trials and used a set of five different delays, leading to five different trial types. These were presented in a pseudo-randomized fashion so that each block of 20 trials used each delay four times. This also meant that a given delay could be presented a maximum of eight times in a row. If rats had performed an omission the protocols were designed so that the rat had to rerun a trial with the same delay. The pseudo-randomization of sample levers in the delayed non-matching to position protocol was also changed compared to before. Specifically, each block of four trials used each sample lever twice. The rats were trained on several protocols with gradually increasing delay durations. The aim of this was to find a delay set where a clear drop in the rats’ success rate could be seen between trials with the shortest and longest delay. For the delayed alternation protocol, the delay sets were as follows: 0, 1, 2, 3, 4 seconds / 0, 1, 3, 6, 9 seconds / 0, 1, 4, 8, 12 seconds / 0, 2, 5, 10, 15 seconds / 0, 4, 8, 12, 16, 20 seconds. During the first test, the age rats progressed from one protocol to another when they had shown above 80% success on three consecutive sessions. During retesting at older ages, this criterion was reduced to rats performing above 80% success on two consecutive sessions. Exceptions to this performance-based criterion were the two last delay sets. Specifically, rats were given three training sessions on the second last delay set, regardless of their success rate. Training on the last delay set continued until rats showed a stable performance, as defined below. The delay sets used for the delayed non-matching to position test were: 0, 1, 2, 3, 4 seconds / 0, 1, 3, 6, 9 seconds / 0, 1, 4, 8, 12 seconds / 0, 2, 5, 10, 15 seconds / 0, 5, 10, 15, 20 seconds / 0, 5, 10, 15, 20, 25 seconds. The same criterions as described above were used for progressing through these delay sets. Notably, the last delay set for both the delayed alternation and the delayed non-matching to position protocol used six delays rather than five. To accommodate this, the number of trials per session and the pseudo-randomization were adjusted. The delayed alternation sessions were set to last 120 trials or 60 minutes. The trials were organized so that each block of 12 trials used each delay twice. For the delayed non-matching to position protocol, the number of trials was initially set to 96, but was reduced to 48 for a large part of the study (all baselines except the ones for the 4 months performance at standard food restriction and both baselines from the 19 months test). The reason for this was that many rats were not motivated enough to perform 96 trials and we sought to minimize differences in possible within-session training effects. The protocol was still set so that each block of four trials used each sample lever twice, while the delays were organized in the same way as the delayed alternation trials. It should be noted that the pseudo-randomization limits described above were not completely reliable. However, they functioned well enough to ensure that rats experienced comparable numbers of each trial type on any given session (+/- 3 trials). In addition, the baselines were constructed from performance over several consecutive sessions, thus minimizing the effect that slight differences in the frequency of a given trial type would have had on the overall performance.

As noted, the rats were trained on the final delay sets until they showed a stable performance. When this was achieved, data from a number of consecutive sessions were used to create the baseline data that was used for detailed analysis. At each test age this baseline data was first gathered while the rats were maintained on the standard food restriction protocol. Afterwards, the food restriction protocol was changed. Rats of both genotypes were continuously given daily sessions through the restriction adjustment. When the alternative food restriction levels had been established and rats were once again performing stably, data for a second performance baseline was gathered. Once the data had been gathered, the rats were once again given free access to food and the test ended.

Both the delayed alternation and the delayed non-matching to position tests are well described in literature [23–29,32]. Our protocols were based on the general consensus and small optimizations of these references.

### Operant conditioning protocol parameters

The operant conditioning system created individual log files for each training session and rat. These log files were run through a series of in-house designed analysis scripts written in R, to obtain a large set of parameters that were used for subsequent analysis.

The number of sessions required to reach the various performance criteria served as a major parameter for evaluating how animals learned the given tasks and progressed through the series of protocols. Success rate (i.e. the percent of trials with successful responses) was calculated differently depending on the protocols used. During the free alternation and the non-matching to position sequence learning protocols, the calculation included all completed trials to give a single success rate value for each session. For protocols where delays were present, separate success rates were calculated for each trial type (i.e. trials with different delays) so that curves plotting success rate against delay durations could be created for each session. These curves served as the main readout of the tests and were used to determine when the rats had reached stable performance on the final delay sets. During testing, the rats’ mean performance on each block of three consecutive sessions was calculated. When statistical analysis showed no significant change between several consecutive session blocks, the rats were considered to have reached stable performance. As noted, the sessions within the blocks where stable performance was found were used for detailed analysis of baseline performance. Although the exact number of sessions included in these analyses varied between baselines, it stayed between 9 and 12 sessions. As noted above, only completed trials (i.e. trials where the rats performed either a correct or incorrect response) were included in success rate calculations. The number and frequency of omission trials (trials where rats failed to perform a head entry or lever push within the set time limit) constituted their own analysis.

The protocols offered several parameters regarding the rats’ latency to perform specific responses. For the free alternation protocol, this primarily included the latency to start trials (measured from the pellet trough light being switched on to the rat entering the pellet trough, triggering lever insertion) and the latency to respond to the inserted levers (measured from lever insertion to lever push). The free non-matching to position protocol included similar parameters, with trial start latency (measured from the pellet trough light being switched on, to the rat entering the pellet trough, triggering insertion of the sample lever), latency to respond to the sample lever (measured from lever insertion to lever push), latency to return to the pellet trough (measured from release of sample lever to the rat entering the pellet trough, triggering the choice step) and the latency to perform a lever response during the choice step (as above, measured from lever insertion to lever push). When delays were added to the protocols, the exact measurement made by some of these parameters were slightly modified and additional parameters were added to ensure a comprehensive analysis of the rats’ behavior. For the delayed non-matching to position protocol the latencies to start trials and respond to levers were measured in the same way as during the free non-matching to position protocol. The latency to return to the pellet trough after responding to the sample lever was, however, replaced by the parameters for the latency to perform the first head entry of the delay (measured from release of sample lever to first entry) and the latency to trigger the choice step (measured from the end of the delay, to the point when rats performed the entry that triggered insertion of both levers). It is important to note that trials with 0 second delays were only included in the latter analysis. For the delayed alternation, the lever response latency was measured in the same way as during the free alternation protocol. However, the trial start latency was now measured from the end of the delay to the point when rats performed the entry that triggered lever insertion. Similarly to the delayed non-matching to position protocol, a measurement for the latency to perform the first head entry of the delay (measured from the pellet trough light being switched on to the point when rats performed the first entry) was added. The distinction of these various parameters is important to consider when comparing the performance between the various protocols. Thus, the trial start latency in the free alternation, free non-matching to position and delayed non-matching to position can be considered a measurement of how fast the rats respond to the light in the pellet trough being switched on. However, in the delayed alternation protocol, this behavior is best described by the latency to perform the first head entry of the delay rather than the trial start latency. Further, the trial start latency of the delayed alternation protocol is closely connected to the rats’ interest in the pellet trough during the delays, and is comparable to the latency to trigger the choice step in the delayed non-matching to position protocol. The lever response latency in the alternation protocols is comparable to the choice lever response latency in the non-matching to position protocols. Finally, the latency to respond to the sample lever and perform the first entry of the delay during the delayed non-matching to position protocol lack direct counterparts in the delayed alternation protocols. Additional parameters were used to investigate the rats’ behavior during delay steps. These measured the mean number of entries and the total time spent inside the pellet trough during delay steps, as well as the mean duration of individual entries. Trials with the longest delays were subjected to further analysis. Specifically, the mean number of head entries performed during discrete segments of these delays was evaluated in order to investigate if the rats’ interest in the pellet trough changed with time. The latency to retrieve reward pellets was investigated for all tests. This was measured from the point of releasing the reinforced lever to entering the pellet trough.

As with the success rate analysis, the parameters described above were only evaluated for completed trials. For alternation protocols, the first trial of the session and the first trial following omissions (i.e. trials where any lever response would be reinforced) were also excluded. Analysis of free alternation and free non-matching to position performance was made over all completed trials. In contrast, separate analysis of trials with different delay durations was performed for most of the parameters in the delayed alternation and delayed non-matching protocols. Analysis was primarily made over all trials regardless of outcome, although separate analyses for successful and failed trials were also performed.

### Video scoring

As noted above, several parameters were used to evaluate the rats’ behavior during delay steps. All these parameters used readouts from the entry sensor in the pellet trough. Interpretations of these parameters can occasionally be difficult, as the signaled number of entries does not always correspond to the actual number of entries. Thus, several videos were recorded during the last test age in order to manually score their behaviors during delays. Video scoring was performed with the Observer XT software (v.12.5.927, Noldus, The Netherlands, Wageningen). The following behaviors were scored during delays:

#### Time spent in pellet trough

This considered all occasions where a rat had anything from its nose to its entire head inside the pellet trough.

#### Time spent in a central position

This considered all occasions where a rat had its head inside the pellet trough. It also included all occasions where a rat was sitting in front of the pellet trough, keeping its head outside, while still appearing to focus on it. In addition, it included occasions where a rat investigated the wall portion that was positioned directly above the pellet trough.

#### Body shifts towards the left or right side

With quite high frequency, the rats would exit the pellet trough to briefly investigate the wall portions to the right or left of the pellet trough, and then return. These body shifts occurred in several different forms. Some were short, and the rat only quickly indicated an interest to either the right or left side. Others were longer and could include both direct investigation of the lever slots or more general investigation of the surrounding wall area. All body shifts, regardless of duration or specific nature, were included in the analysis. During analysis, separate scores were given for shifts to the left and right side.

All scoring focused on noting start and stop point of each occasion where a rat displayed the above mentioned behaviors. The logs from the Observer XT software were later combined with the log files from the operant conditioning system. These were run through in-house designed R scripts to obtain detailed analysis. Through this, the number of behavioral episodes, their mean duration and the total time spent on the different behaviors could be evaluated for individual delay steps. The estimated amount of time spent investigating other parts of the operant conditioning boxes during delays was also calculated. These calculations primarily considered time spent investigating the back wall as well as the back halves of the left and right wall of the operant conditioning box. The calculations were based on the total time for all behaviors noted above and the known delay durations. The body shifts were initially scored as being made either to the left or right side of the pellet trough, although they were later relabeled depending on if they were made towards the correct or incorrect lever, or if they were made towards the lever that the rats eventually responded to. The latter was initially used to assess whether the body shifts at all constituted a form of strategy. It was further used to evaluate how rats established, maintained and shifted focus during delays. For this, the rats were considered to have established a focus for one particular lever based on their first body shift during a given delay step. The rats were then considered to have maintained or changed it, if the last body shift during the delay step was made towards the same or the opposite side, respectively. Thus, the focus behavior during each delay step was classified as having no focus (no body shifts occurred), established focus (only one body shift occurred), maintained focus (first and last body shifts of delay were made towards the same side) or changed focus (first and last body shifts of delay were made towards different sides). Further scores were made to evaluate if the initial focus had been made towards the correct or incorrect lever. Finally, specific analysis was made for trials with the longest delay steps. For this, the relative amount of time spent around the correct lever during segments of the delay was analyzed separately for successful and non-successful trials. In addition to the behaviors that were scored during delays, the rats’ behavior during lever responses was also investigated. Specifically, it was noted if rats had responded to the chosen lever without showing any interest in the other lever (direct responses), if the rats first headed for one lever but changed their mind and ultimately responded to the other lever (corrections) or if the rats went back and forth between the two levers a few times before finally deciding on one (uncertain responses). In addition to investigating the frequency of the different behaviors, theoretical success rate curves were created to assess the importance of the corrections. For this, the hypothetical results of rats responding according to the lever they first showed interest in during correction trials was considered.

### Statistical analysis

All statistical analyses were conducted using GraphPad Prism v.6.01 (GraphPad Software, San Diego California USA, http://www.graphpad.com).

The results from most parameters were investigated with different types of two-way repeated measures ANOVAs. Most of these were aimed at investigating genotype differences, and thus focused on data where genotype was used as the non-repeated factor, while either age, delay duration, type of baseline or specific protocol step served as the repeated factor. Certain analyses, however, were performed within genotype groups, and aimed at investigating performance differences between baselines at different ages, baselines at different food restriction protocols or performance on successful or failed trials. All these analyses used two-way repeated measures ANOVAs where both delay and the other given factor were considered to be repeated factors. This kind of ANOVA was also used when evaluating if rats had reached a stable performance baseline. The results from video scoring the frequency of different behaviors during delay and lever steps of the delayed alternation and delayed non-matching tests were analyzed with separate two-way ANOVAs for the different behaviors. As above, these used genotype as the non-repeated factor and delay as the repeated factor. Sidak’s multiple comparison *post-hoc* test was used to follow up any significant effects found in the two-way ANOVAs. The number of sessions required to progress through the set of delayed alternation and delayed non-matching to position protocols with gradually increasing delay durations was analyzed in several single comparisons between WT and BACHD rats. For these, t-test, t-test with Welch’s correction or Mann-Whitney test were used, depending on the data’s apparent distribution.

During testing there were occasionally rats that fell ill and had to be sacrificed. Thus, the n of the analyses changed as follows. For the delayed alternation group, 2–4 months (WT:12, BACHD: 12), 4–9 months (WT: 12, BACHD: 12), 12–14 months (WT: 11, BACHD: 11) and 17–19 months (WT: 9, BACHD: 8 during standard food restriction protocol, 7 during alternative food restriction protocol). For the delayed non-matching to position group, 2–4 months (WT:12, BACHD: 12), 4–9 months (WT: 12, BACHD: 12), 12–14 months (WT: 12, BACHD: 12) and 17–19 months (WT: 12 during standard food restriction protocol, 11 during alternative food restriction protocol, BACHD: 11). Video scored behavior concerned (WT: 9, BACHD: 7) and (WT: 11, BACHD: 11) for the alternation and non-matching tests, respectively. Age development analyses excluded data from animals that were not assessed at all ages. No other exclusion criteria were used. As described in the Results section, there was very rarely any clear effect of age found on the various parameters. Thus, for most baseline parameters the analysis was performed on the mean performance of all evaluated ages to maintain an n of 12.

Alpha for all analyses was set to 0.05.

## Results

### Survival

Most rats remained healthy through the entire duration of the tests, although some rats had to be sacrificed due to illness. All in all, three WT and five BACHD rats were sacrificed from the delayed alternation group, and one rat of each genotype was sacrificed from the delayed non-matching to position group. In most cases, the illnesses concerned tumors. The change in n for the different groups is described in detail in the Material and Methods section.

### Basic operant conditioning protocols

There were no consistent or overt performance differences between BACHD and WT rats during habituation, CRF training, forced alternation training or non-matching to position sequence training (data not shown). All rats quickly progressed through their specific set of protocols, and rarely required more than a single session per step. BACHD rats were during CRF training occasionally found to be slower than WT rats when returning to the reinforced lever after retrieving a pellet reward (data not shown).

### Free and delayed alternation performance

During the first three test ages, BACHD and WT rats completed comparable numbers of trials on all of the investigated protocols described below. During the 19-month test age, BACHD rats tended to complete fewer trials than WT rats on the protocol with the final delay set, although the difference did not reach statistical significance (data not shown). There were at no point any differences concerning the ratio of completed Left-Right and Right-Left alternations between WT and BACHD rats (data not shown).

**Fig 3:**
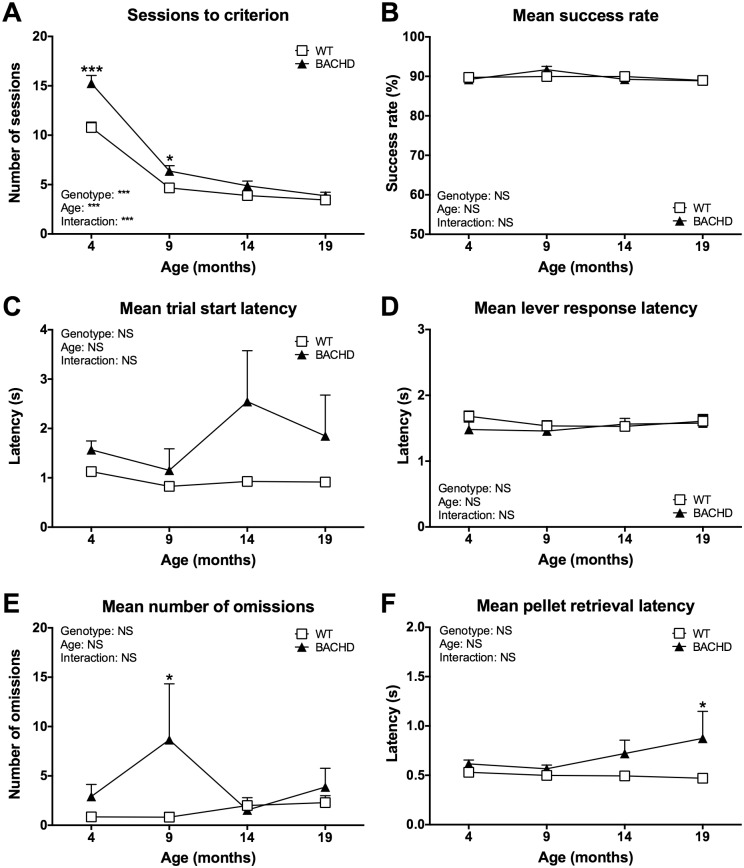
Age development of free alternation performance. The graphs show the main readouts of the free alternation protocol over the four test ages. (**A**) shows the number of training sessions required for reaching criterion. (**B**)–(**F**) show the mean performance of rats during sessions where their success rate was at criterion level. Curves show group mean plus standard error. Results from two-way repeated measures ANOVA are shown inside the graphs. Results from *post-hoc* analysis are indicated in case significant genotype differences were found. * (*P* < 0.05) ** (*P* < 0.01) *** (*P* < 0.001).

The number of sessions needed to reach criterion on the free alternation protocol decreased with repeated testing for rats of both genotypes (Fig 3A) (age effect: F_(3,45)_ = 253.8, *P* < 0.001). BACHD rats required more sessions compared to WT rats during the first two test ages, as indicated by a significant genotype effect (F_(1,15)_ = 19.15, *P* < 0.001), genotype x age interaction effect (F_(3,45)_ = 10.96, *P* < 0.001) and post-test results (4 months: *P* < 0.001; 9 months: *P* < 0.05) (Fig 3A). During criterion-level performance, there were no consistent differences between WT and BACHD rats in terms of success rate (Fig 3B), trial start latency (Fig 3C), lever response latency (Fig 3D) or number of omissions (Fig 3E). BACHD rats did, however, become progressively slower at retrieving the reward pellets, resulting in them showing significantly longer latencies compared to WT rats at the last test age (Fig 3F) (post-test result at 19 months: *P* < 0.05).

Most rats reliably reached the performance criterion on each delayed alternation protocol, and thus progressed properly through the series of delay sets. A total of four BACHD rats did not consistently reach each performance criterion and would occasionally get stuck on a particular delay set (two to three out of the four rats at each test age). The rats would in these cases show no clear indication of improving their performance, despite being given extensive training (up to ten sessions with arguably stable performance). Their performance typically remained close to criterion, being just above or below it on more or less alternating sessions. These rats were still allowed to continue through the series of delayed alternation protocols, as they were deemed to simply have reached their maximum performance. The rats were, however, excluded from the analysis of the number of sessions required to progress through all the protocols. This analysis showed that rats of both genotypes required a high number of sessions during the first test age, but then dropped to a relatively stable level during retesting (S1 Fig). BACHD rats needed significantly more sessions than WT rats to progress through the series of delayed alternation protocols during the first three test ages, although the phenotype was strongest during the first test age (S1 Fig) (single comparisons: 4 months: *P* < 0.001; 9 months: *P* < 0.05; 14 months: *P* < 0.05).

**Fig 4:**
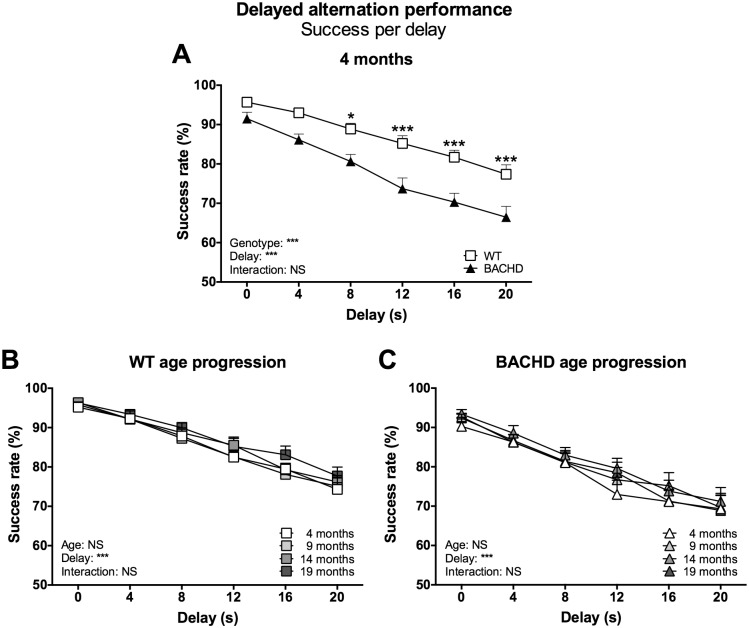
Success rate per delay in the delayed alternation test. The graphs show the success rate on trials preceded by delays of different durations in the delayed alternation test. (A) shows the stable baseline performance of rats maintained on the standard food restriction protocol at four months of age. (B) and (C) show the age progression of performance for WT and BACHD rats. Curves display group mean plus standard error. Results from two-way repeated measures ANOVA are shown inside the graphs. For (A), results from *post-hoc* analysis are indicated in case significant genotype differences were found. * (*P* < 0.05) ** (*P* < 0.01) *** (*P* < 0.001).

The main parameter of interest for the delayed alternation test concerned the success rate on the different trial types. Analysis of this parameter showed that rats of both genotypes maintained a high success rate when trials were preceded by a delay of zero seconds, but dropped as the delay duration increased (Fig 4A for 4-month data, S2 Fig for 9-, 14- and 19-month data) (delay effect at 4 months: F_(5,110)_ = 66.14, *P* < 0.001; 9 months: F_(5,110)_ = 81.59, *P* < 0.001; 14 months: F_(5,100)_ = 103.4, *P* < 0.001; 19 months: F_(5,75)_ = 70.50, *P* < 0.001). BACHD rats performed generally worse than WT rats at all investigated ages, as indicated by significant genotype effects for all baseline comparisons (genotype effect at 4 months: F_(1,22)_ = 19.99, *P* < 0.001; 9 months: F_(5,110)_ = 13.66, *P* < 0.01; 14 months: F_(5,20)_ = 11.20, *P* < 0.01; 19 months: F_(5,15)_ = 8.79, *P* < 0.01) without statistically significant genotype x delay interaction. Still, trends and *post-hoc* analysis indicated that the reduced success rate among BACHD rats was more pronounced on trials preceded by longer delays. Performance and phenotypes did not notably change with age when rats were retested (Fig 4B and 4C, S2 Fig).

**Fig 5:**
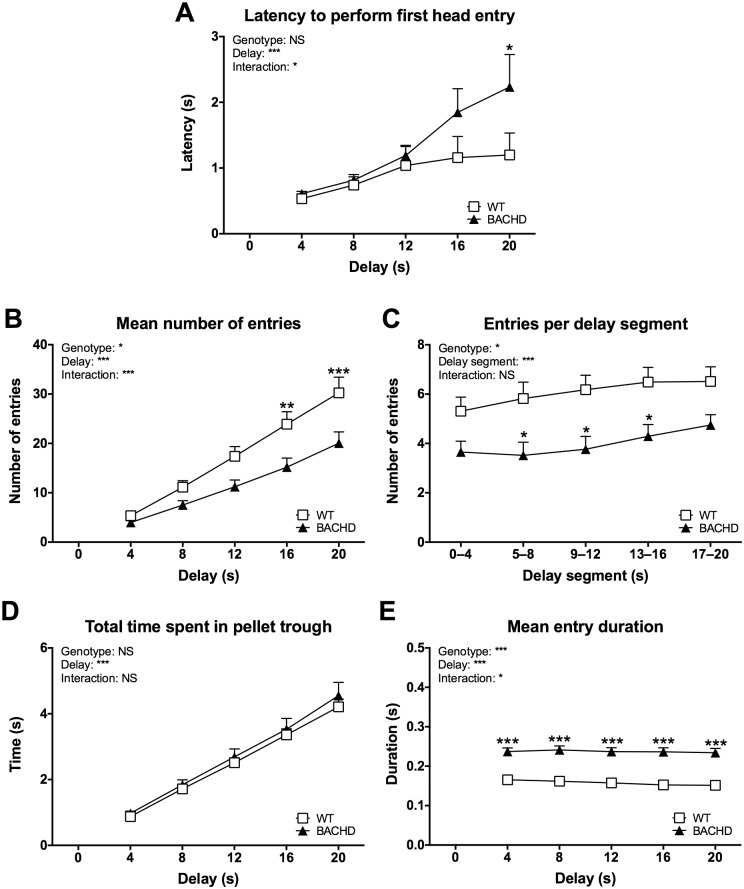
Head entry behavior during delays of the delayed alternation protocol. The graphs show several aspects of head entries made into the pellet trough during delay steps of the delayed alternation test. Curves were created based on the overall performance at all test ages, as no significant change with age was found for the parameters. (C) concerns the 20-second delay step. Graphs indicate group mean plus standard error. Results from two-way repeated measures ANOVA are shown inside the graphs. Results from *post-hoc* analysis are indicated in case significant genotype differences were found. * (*P* < 0.05) ** (*P* < 0.01) *** (*P* < 0.001).

Several additional parameters concerning delayed alternation performance were investigated. One set of parameters concerned the entries made into the pellet trough during delays (Fig 5). These parameters did not appear to change with age and therefore, although initial analyses were made for individual test ages, only mean performance over all test ages is displayed and discussed here. The latency to perform the first head entry during the delay increased with delay duration (Fig 5A) (delay effect: F_(4,88)_ = 13.41, *P* < 0.001). The ANOVA did not reveal an overall genotype difference. However, BACHD rats were slower than WT rats during the longest delay step, as indicated by a significant genotype x delay interaction (F_(4,88)_ = 2.763, *P* < 0.05) as well as a significant genotype difference in *post-hoc* analysis of that data point (head entry latency at 20 months: *P* < 0.05). The number of entries made during delays increased with delay duration (Fig 5B) (delay effect: F_(4,88)_ = 152.4, *P* < 0.001). BACHD rats made generally fewer entries compared to WT rats (genotype effect: F_(1,22)_ = 6.715, *P* < 0.05), although the phenotype was more pronounced during longer delays, as indicated by a significant genotype x delay interaction effect (F_(4,88)_ = 7.554, *P* < 0.001) and significant genotype differences in *post-hoc* analyses (16-second delay: *P* < 0.01, 20-second delay: *P* < 0.001). To gain further insight into the behavior, the number of entries made during segments of the 20-second delay was analyzed (Fig 5C). This indicated that BACHD rats made fewer entries than WT rats on all segments of the delay (genotype effect: F_(1,22)_ = 7.852, *P* < 0.05, post-test result: *P* < 0.05 for the 5–8, 9–12, and 13–16 seconds delay segments). However, WT and BACHD rats still spent comparable amounts of time inside the pellet trough (Fig 5D), as BACHD rats made generally longer entries compared to WT rats (Fig 5E) (genotype effect: F_(1,22)_ = 33.34, *P* < 0.001, post-test result: *P* < 0.001 for all delays).

During the 4-month test age, there was no difference between WT and BACHD rats regarding their trial start latencies (S3A Fig) or number of trial start omissions (S3B Fig). However, a peculiar performance difference developed during retesting. Specifically, BACHD rats showed longer trial start latencies compared to WT rats on trials that were preceded by intermediate delays, but not on trials preceded by 0- or 20-second delays (S3C Fig) (genotype difference in *post-hoc* analysis 4-second delay: *P* < 0.05, 8-second delay: *P* < 0.001, 12-second delay: *P* < 0.01). The behavioral basis for this phenotype was discovered during video scoring and is discussed further below. Specifically, it was found that BACHD rats frequently turned away from interactive wall in order to drink. The same behavior also caused BACHD rats to perform a higher number of trial start omissions than WT rats on trials preceded by short delays (S3D Fig) (genotype difference in *post-hoc* analysis 0-second delay: *P* < 0.01, 4-second delay: *P* < 0.001, 8-second delay: *P* < 0.05).

There were no overt differences in lever response latencies between the genotypes, although BACHD rats appeared to be a bit slower than WT rats at responding during trials preceded by a 0-second delay (S4A Fig). Finally, BACHD rats were slower than WT rats at retrieving the reward pellets (S4B Fig). The phenotype became more apparent with age, and the age progression analysis shown in S4 Fig only found a significant phenotype during the last test age (genotype difference in *post-hoc* analysis 19 months: *P* < 0.05). It should, however, be noted that single comparisons at each test age reliably revealed a significant genotype difference (data not shown).

The results described above were from analyses that included all completed trials, i.e. both successful and failed trials (although excluding omitted ones). Analysis of each parameter was, however, also performed based on trial outcome (S5 Fig). This was done to evaluate, if the BACHD rats’ lower success rate is connected to the other noted behavioral differences. Among BACHD rats, failed trials were preceded by delays with slightly fewer entries (S5A Fig) (trial type difference in *post-hoc* analysis 8-second delay: *P* < 0.05, 12-second delay: *P* < 0.05, 16-second delay: *P* < 0.01, 20-second delay: *P* < 0.01) and slightly less time spent in the pellet trough (S5B Fig) (trial type difference in *post-hoc* analysis 8-second delay: *P* < 0.05, 16-second delay: *P* < 0.05, 20-second delay: *P* < 0.05). However, these differences were small and not consistently present at all individual test ages, and thus unlikely to be of major importance. Trial start latencies (S5C Fig) and lever response latencies (S5D Fig) instead appeared to have stronger impact on the trial outcome. On trials preceded by short delays, failing appeared to be related to long trial start latencies for BACHD rats (trial type difference in *post-hoc* analysis 0-second delay: *P* < 0.001, 4-second delay: *P* < 0.001, 8-second delay: *P* < 0.05), while being related to long lever response latencies for WT rats (trial type difference in *post-hoc* analysis 0-second delay: *P* < 0.001, 4-second delay: *P* < 0.001).

Changing the food restriction protocol so that the WT rats’ food restriction level increased from 85% (standard food restriction) to 95% (alternative food restriction) did not markedly change the rats’ behavior. They still completed all trials of the sessions and performed comparable number of Left-Right and Right-Left alternations (data not shown). The success rate per delay remained completely unchanged by the adjustment of food restriction at all ages (S6 Fig). The shift also did not have any overt effects on the other parameters of the delayed alternation protocol (S7 Fig). Still, trial start latencies (S7C Fig) and lever response latencies (S7D Fig) appeared to become longer after adjustment (specific effect among WT rats being changed to a lower restriction level, not seen for BACHD rats during the extended training) (food restriction effect on trial start latency in WT rats: F_(1,11)_ = 11.91, *P* < 0.01; food restriction effect on lever response latency in WT rats: F_(1,11)_ = 9.55, *P* < 0.05). For the trial start latencies, the change primarily concerned the intermediate delays (food restriction difference in *post-hoc* analysis 4-second delay: *P* < 0.001, 8-second delay: *P* < 0.001, 12-second delay: *P* < 0.001; 16-second delay: *P* < 0.01). Despite this, the aforementioned genotype difference in trial start latency largely remained the same (data not shown). The number of omissions was affected both by the motivational shift due to food restriction adjustment and by extended training (S8A Fig). Specifically, WT rats performed more omissions, while BACHD rats performed fewer ones, resulting in a significant genotype x baseline interaction effect (F_(1,22)_ = 15.42, *P* < 0.001). The change among BACHD rats appeared to be connected to a slightly lower omission rate on trials preceded by no delay (S8B Fig) (baseline difference in *post-hoc* analysis 0-second delay: *P* < 0.01), while the change among WT rats concerned an increase in their omission rates on all other trial types (baseline difference in *post-hoc* analysis: *P* < 0.001 for 4-, 8-, 12-, 16-, and 20-second delays). Despite these changes, the initial phenotype of BACHD rats performing more omissions than WT rats was not resolved (data not shown).

### Free and delayed non-matching to position performance

BACHD rats tended to complete fewer trials than WT rats (data not shown), although this could not be investigated in detail, as the session duration was adjusted so that rats of both genotypes would complete comparable numbers of trials. Despite these efforts, BACHD rats were found to complete significantly fewer trials than WT rats during the 19-month test age (data not shown). At that point, BACHD rats completed on average 84 trials, while WT rats completed 96 trials. There was at no point any difference concerning the ratio of completed Left-Right and Right-Left trials between WT and BACHD rats (data not shown).

**Fig 6:**
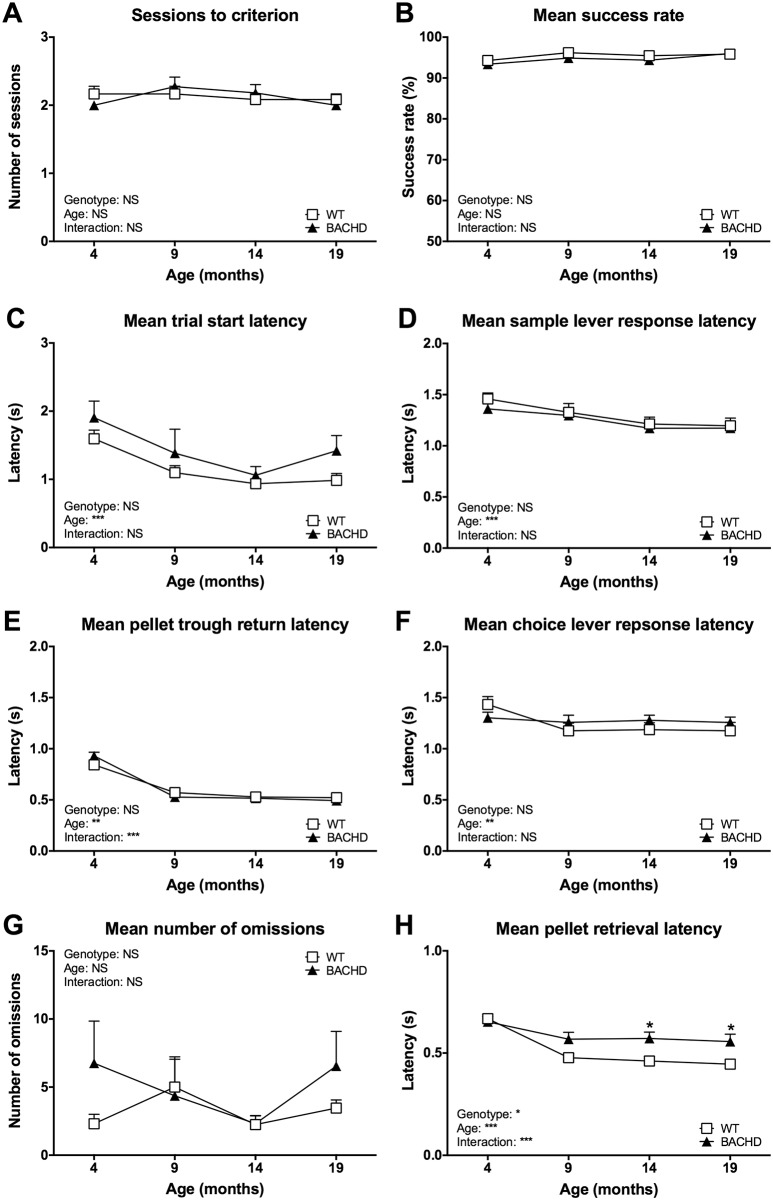
Age development of free non-matching to position performance. The graphs show the main readouts of the free non-matching to position protocol over the four test ages. (A) shows the number of training sessions required for reaching criterion. (B)–(H) show the mean performance of rats during sessions where their success rate was at criterion level. Session to criterion data was corrected for the change in criterion between the first test age and retesting. Curves show group mean plus standard error. Results from two-way repeated measures ANOVA are shown inside the graphs. Results from *post-hoc* analysis are indicated in case significant genotype differences were found. * (*P* < 0.05) ** (*P* < 0.01) *** (*P* < 0.001).

The free non-matching to position protocol was, in contrast to the free alternation, very easy for the rats to learn. Thus, after the initial response sequence training and upon retesting at older ages, most rats performed at criterion level from the first session onwards. This resulted in rats needing very few sessions to reach the performance criterion, and no difference was found between the genotypes concerning this parameter at any age (Fig 6A). During performance at criterion level, there were no differences regarding success rate (Fig 6B), trial start latency (Fig 6C), sample lever response latency (Fig 6D), pellet trough return latency (Fig 6E), choice lever response latency (Fig 6F) or number of omissions (Fig 6G). BACHD rats were, however, found to be slower than WT rats at retrieving the reward pellets (Fig 6H). This phenotype was most pronounced during the last two ages and not present during the first test age, as indicated by a significant genotype effect (F_(1,21)_ = 5.265, *P* < 0.05), significant genotype x age interaction effect (F_(3,63)_ = 6.338, *P* < 0.001) and results from *post-hoc* analysis (significant genotype differences at 14 and 19 months: *P* < 0.05).

Most rats progressed properly through the delayed non-matching to position protocols with increasing delay durations by reaching the performance criterion of each protocol. There were, however, a total of three BACHD rats that did not reliably manage to reach each criterion and thus would occasionally get stuck at a particular delay set despite extensive training. The rats did not consistently show the problems, meaning that at each given test age there were between zero and three out of those three BACHD rats that did not manage all performance criteria. The rats were handled like the ones in the delayed alternation. Thus, they were allowed to continue through the series of protocols, were part of the main performance analysis, but not the specific analysis concerning the number of sessions required to progress through the series of delay sets. This sessions to criterion analysis indicated that rats needed a relatively stable number of sessions to reach the final delay step, and the two genotypes required similar numbers of sessions at all test ages (S9 Fig).

**Fig 7:**
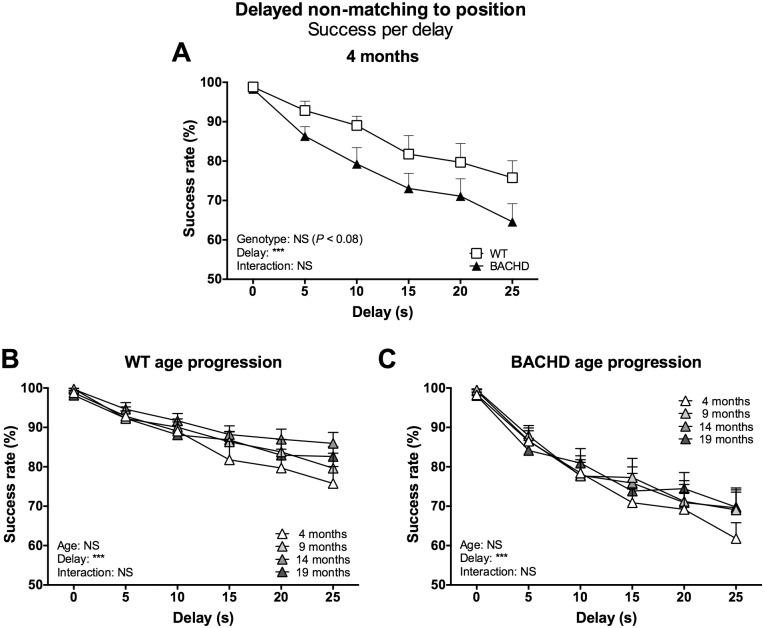
Success rate per delay in the delayed non-matching to position test. The graphs show the success rate on trials with delays of different durations in the delayed non-matching test. (A) shows the stable baseline performance of rats maintained on the standard food restriction protocol at four months of age. (B) and (C) show the age progression of performance for WT and BACHD rats, respectively. Curves display group mean plus standard error. Results from two-way repeated measures ANOVA are shown inside the graphs. For (A), results from *post-hoc* analysis are indicated in case significant genotype differences were found. * (*P* < 0.05) ** (*P* < 0.01) *** (*P* < 0.001).

The main parameter of interest was once again the success rate on trials with different delay durations. Analysis of this parameter showed that rats of both genotypes maintained a high, close to 100%, success rate on trials with a 0-second delay, but dropped as the delay duration increased (Fig 7A for 4-month data, S10 Fig for 9-, 14- and 19-month data) (delay effect at 4 months: F_(5,110)_ = 40.10, *P* < 0.001; 9 months: F_(5,110)_ = 32.51, *P* < 0.001; 14 months: F_(5,110)_ = 35.76, *P* < 0.001; 19 months: F_(5,105)_ = 48.53, *P* < 0.001).

While there was no difference between the genotypes’ performance on trials with 0-second delays, the BACHD rats’ success rate dropped more than WT rats’ on trials with 5- and 10-second long delays. Interestingly, the two genotypes appeared to show a comparable decline in success rate for trials with longer delays. Ultimately, while WT rats showed reasonably linear drops in success rate with increasing delays, BACHD rats appeared to show a biphasic curve. Still, statistical analysis failed to reliably detect significant differences in the rats’ performance. No differences were found during the first two test ages, while the last two presented both significant genotype effects (14 months: F_(1,22)_ = 11.01, *P* < 0.01; 19 months: F_(1,21)_ = 4.95, *P* < 0.05) and genotype x delay interaction effects (14 months: F_(5,110)_ = 4.49, *P* < 0.001; 19 months: F_(5,105)_ = 4.23, *P* < 0.01). As this did not appear to be due to either of the genotypes changing their behavior with repeated testing (Fig 7B and 7C), and as the performance during the first test age still showed a quite strong genotype effect trend (F_(1,22)_ = 3.44, *P* = 0.08), this ultimately indicated a stable but discrete phenotype sensitive to small variations in the recorded data. The notion of a biphasic success rate curve for BACHD rats was supported by the fact that the genotype x delay interaction effect was also found when the analysis was limited to trials with 0- to 10-second delays (data not shown), while analysis of trials with 10- to 25-second delays did not reveal a significant genotype x delay but only an overall genotype effect (data not shown). Thus, the phenotype was dependent on the presence of delays, although the extent of impairment was not directly related to their duration.

**Fig 8:**
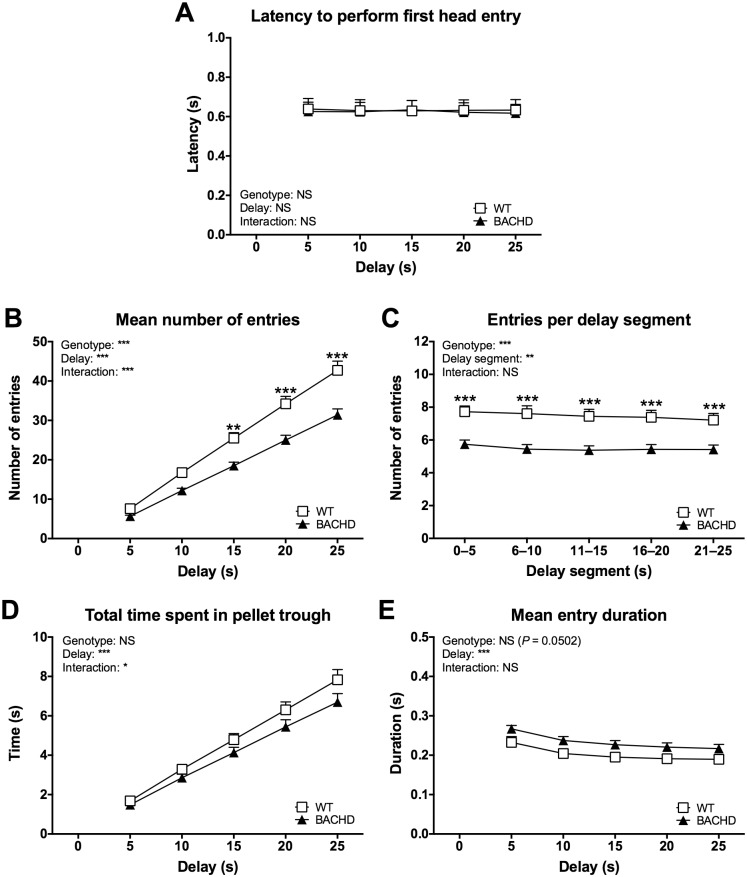
Head entry behavior during delays on the delayed non-matching to position protocol. The graphs show several aspects of head entries made into the pellet trough during delay steps of the delayed non-matching to position test. Curves were created based on the overall performance on all test ages, as no consistent change with age was found for the parameters. (C) concerns the 25-second delay step. Graphs indicate group mean plus standard error. Results from two-way repeated measures ANOVA are shown inside the graphs. Results from *post-hoc* analysis are indicated in case significant genotype differences were found. * (*P* < 0.05) ** (*P* < 0.01) *** (*P* < 0.001).

As with the delayed alternation protocol, several additional parameters concerning delayed non-matching to position performance were investigated. Once again, one set of parameters concerned the entries made into the pellet trough during delay periods (Fig 8). Performance on these parameters did not appear to change with age and therefore, although initial analyses were made for individual test ages, only the mean performance over all test ages is displayed and discussed here. The latency to perform the first head entry of the delay remained stable with delay duration and did not differ between WT and BACHD rats (Fig 8A). The number of entries made during delays increased with delay duration (Fig 8B) (delay effect: F_(4,88)_ = 613.5, *P* < 0.001). BACHD rats made generally fewer entries compared to WT rats (genotype effect: F_(1,22)_ = 17.57, *P* < 0.001), although the phenotype was more pronounced during longer delays, resulting in a significant genotype x delay interaction effect (F_(4,88)_ = 14.62, *P* < 0.001) and significant genotype differences in *post-hoc* analyses (15-second delay: *P* < 0.01, 20-second delay: *P* < 0.001, 25-second delay: *P* < 0.001). To gain further insight, the number of entries made during segments of the 25-second delay was analyzed (Fig 8C). This once again indicated that BACHD rats made fewer entries than WT rats throughout the delay, rather than during specific parts of it (genotype effect: F_(1,22)_ = 17.00, *P* < 0.001; genotype difference in *post-hoc* analyses: *P* < 0.001 for all delay segments). Through this, BACHD rats ended up spending slightly less time inside the pellet trough during the delays compared to WT rats (Fig 8D). Still, this phenotype was weak and only resulted in a significant genotype x delay interaction effect (F_(4,88)_ = 2.58, *P* < 0.05), but not in an overall genotype effect or significant differences in *post-hoc* analyses. This was likely due to the strong trend indicating that BACHD rats made generally longer head entries compared to WT rats (Fig 8E) (genotype effect: F_(1,22)_ = 4.29, *P* = 0.0504).

In contrast to the trial start latency during the delayed alternation protocol, the latency to trigger the choice step in the delayed non-matching to position test did not noticeably change with delay duration (S11A Fig). Both genotypes showed similar, and very short, latencies to trigger the choice step. In connection, neither genotype performed frequent omissions at this point of the protocol (S11B Fig). Rather, the main omission type in the delayed non-matching to position test concerned the trial starts, where BACHD rats performed slightly more omissions compared to WT rats (S11C Fig) (post-test result: *P* < 0.05). The latency to start individual trials was not different between WT and BACHD rats at any of the investigated ages (S12 Fig). Response latency to sample levers did not notably change with delay duration or differ between the genotypes (S13A Fig). Response latencies during choice steps were affected by delay duration, with rats of both genotypes being slightly slower at responding during trials with 0-second delays compared to all other delay durations (S13B Fig) (delay effect: F_(5,110)_ = 11.88, *P* < 0.001). Regardless of this effect, BACHD and WT rats showed identical choice response latencies. For both genotypes, response latencies during the choice step were generally shorter than response latencies to sample levers (S13C and S13D Fig) (trial step effect WT: F_(1,11)_ = 8.501, *P* < 0.05; trial step effect BACHD: F_(1,11)_ = 9.547, *P* < 0.05). Finally, BACHD rats were generally slower at retrieving the reward pellets during the delayed non-matching to position test (S14A Fig) (genotype effect: F_(1,21)_ = 6.638, *P* < 0.05). Although the exact retrieval latency differed with age (age effect: F_(3,63)_ = 5.845, *P* < 0.01), there was no significant genotype x age effect, suggesting that the phenotype was similarly apparent at all ages. Pellet retrieval and pellet trough return are two behaviors that depend on comparable motoric aspects. As noted, WT and BACHD rats performed similarly on the former parameter, but differed on the latter. A direct comparison of these two parameters suggested that BACHD rats showed similar latencies for both behaviors (S14B Fig). In contrast, WT rats were faster when they retrieved reward pellets, compared to when they were returning to the pellet trough after responding to the sample lever. This discrepancy resulted in a significant genotype x protocol step interaction effect (F_(1,22)_ = 5.205, *P* < 0.05).

The results described above were from analyses that included all completed trials, i.e. both successful and failed trials (although excluding omitted ones). Analysis of each parameter was, however, also performed based on trial outcome. This was used to evaluate if the noted behavioral differences were connected to the BACHD rats’ lower success rate (S15 and S16 Figs). For WT rats (S15 Fig), failure on trials without delays was related to longer trial start latencies (S15A Fig) (trial type difference in *post-hoc* analysis of 0-second trials: *P* < 0.001), pellet trough return latencies (S15C Fig) (trial type difference in *post-hoc* analysis of 0-second trials: *P* < 0.001), triggering of choice steps (S15F Fig) (trial type difference in *post-hoc* analysis of 0-second trials: *P* < 0.01) and choice lever responses (S15G Fig) (trial type difference in *post-hoc* analysis of 0-second trials: *P* < 0.001). Similar results were seen when analyzing BACHD rats (S16 Fig) (trial type difference in *post-hoc* analysis of 0-second trials: Trial start latency: *P* < 0.05; Pellet trough return latency: *P* < 0.001; Latency to trigger choice step: *P* < 0.01; Choice lever response latency: *P* < 0.001), although the effects appeared to be less pronounced for all parameters except for the choice lever response latency. Importantly, the number of entries and total time spent in the pellet trough during delays were not clearly connected to trial outcome for either genotype (S15D and S15E, S16D and S16E Figs).

Changing the food restriction protocol so that WT rats increased from 85% (standard restriction) to 95% (alternative restriction) of their free-feeding body weight did not markedly change the behavior of the rats. The aforementioned slight difference in the number of completed trials was resolved due to WT rats performing fewer trials after changing the food restriction protocol (data not shown). Rats still completed comparable numbers of Left-Right and Right-Left trials (data not shown). Success rate per delay remained completely unaffected by the change of food restriction protocol at all ages (S17 Fig). The shift did also not have any overt effects on the other parameters of the delayed non-matching to position protocol (S18 Fig). Small increases in the sample lever response latency (S18B Fig), the time spent in the pellet trough (S18E Fig) and the choice lever response latency (S18G Fig) were found. However, similar changes were seen among BACHD rats that were given extended training on the protocol (S19 Fig), suggesting that the changes were not necessarily related to a shift in motivation due to the change in food restriction protocol. The number of trial start omissions made during the test sessions was, however, specifically affected. While BACHD rats typically performed more omissions than WT rats during the initial baselines, this phenotype was resolved when WT rat were maintained on the alternative restriction protocol (S20 Fig). This was due to WT rats performing more trial start omissions than during the initial baselines, while BACHD rats remained unchanged (baseline effect: F_(1,22)_ = 7.29, *P* < 0.05; interaction effect: F_(1,22)_ = 5.90, *P* < 0.05). Other omission types were not notably affected, neither by the motivational change nor the extended training (data not shown).

### Video scoring

As noted, video recordings were made during baseline performance of both the delayed alternation and delayed non-matching test, at the 17–19-month test age. During this, several videos of full training sessions were gathered for each animal. For the delayed non-matching to position test, a single video from each rat was selected for video scoring. Video selection was made so that the performance on the selected sessions (with regard to the parameters presented above) was comparable to the overall baseline. Video analysis of delayed alternation performance was more elaborate. Initial investigation of the rats’ behavior during the test revealed that the BACHD rats frequently turned away from the interactive wall of the conditioning chamber to drink water. In order to focus the analysis on other types of behavior, trials where the rats had been drinking were excluded. To still get a comprehensive analysis that covered a full test session (i.e. 120 trials), data from several separate sessions were combined. Importantly, the gathered data set still recapitulated most of the phenotypes mentioned in the previous sections, including the lower success rate and lower number of head entries during delays seen among BACHD rats (data not shown). The increased number of omissions and longer trial start latencies seen for BACHD rats on trials with intermediate delays were, however, no longer present (data not shown), concluding that the drinking behavior was the underlying cause.

**Fig 9:**
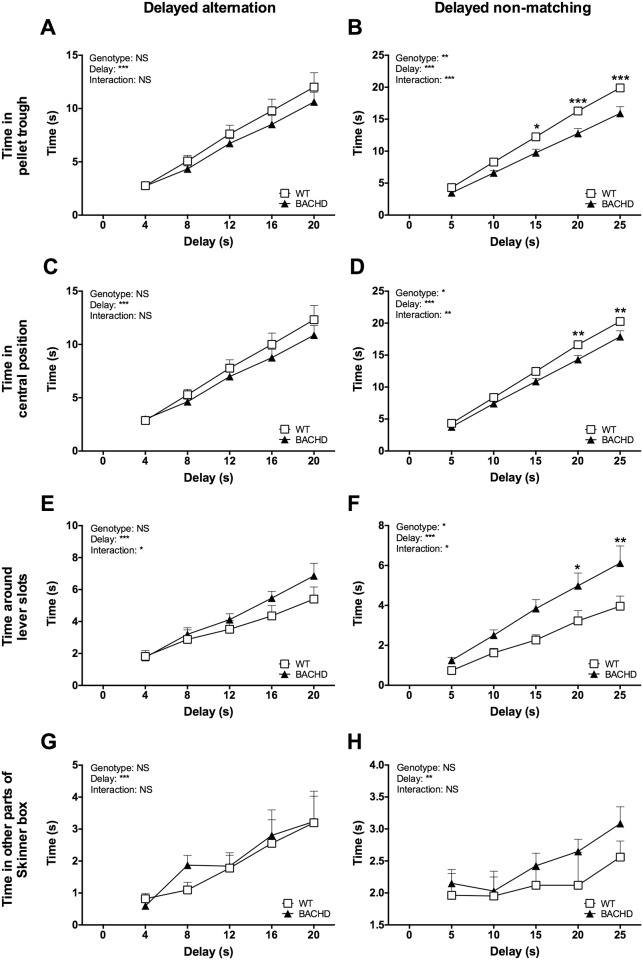
Time spent in different parts of the Skinner boxes during delay steps. The graphs show the time spent in different parts of the Skinner boxes during delays in the delayed alternation and delayed non-matching to position tests, as measured by video scoring. Specific details regarding the data and scoring method is available in the Material and Methods section. Graphs indicate group mean plus standard error. Results from two-way repeated measures ANOVA are shown inside the graphs. Results from *post-hoc* analysis are indicated in case significant genotype differences were found. * (*P* < 0.05) ** (*P* < 0.01) *** (*P* < 0.001).

Video scoring of both tests indicated that the mean amount of time spent inside the pellet trough, in a central position, around the lever slots and in other parts of the operant conditioning chambers increased with delay duration (Fig 9). In the delayed alternation test, BACHD rats appeared to spend slightly less time than WT rats inside the pellet trough (Fig 9A) and in a central position (Fig 9C), while spending more time than WT rats around the levers (Fig 9E). These trends were, however, not strong and with the exception of a significant genotype x delay interaction regarding the time spent around the lever slots (F_(4,56)_ = 2.54, *P* < 0.05), there were no significant effects. The results from the delayed non-matching test showed similar behavioral differences between the genotypes, although much more pronounced. There, BACHD rats spent significantly less time than WT rats both inside the pellet trough (Fig 9B) (genotype effect: F_(1,20)_ = 13.63, *P* < 0.01) and in a central position (Fig 9D) (genotype effect: F_(1,20)_ = 7.58, *P* < 0.05) during delays. Both post-tests and genotype x delay interaction effects indicated that the phenotype was more apparent in trials with longer delays (interaction effect pellet trough: F_(1,20)_ = 7.80, *P* < 0.001; interaction effect central position: F_(4,80)_ = 4.01, *P* < 0.01). BACHD rats also spent significantly more time than WT rats investigating the wall portions around the lever slots during the delayed non-matching protocol (genotype effect: F_(1,20)_ = 6.83, *P* < 0.05). The phenotype was once again more pronounced for trials with longer delays, as indicated by post-tests and a genotype x delay interaction effect (interaction effect: F_(4,80)_ = 2.57, *P* < 0.05). The phenotype was primarily due to the BACHD rats performing a higher number of body shifts towards the different lever slots, while the duration of these body shifts were comparable between the genotypes (data not shown). There were no differences between the genotypes concerning the amount of time they spent in other compartments of the conditioning boxes in either test (Fig 9G and 9H). In the alternation test, there was no difference between genotypes regarding the mean duration of head entries, while BACHD rats were found to make shorter entries compared to WT rats during the delayed non-matching test (data not shown).

**Fig 10:**
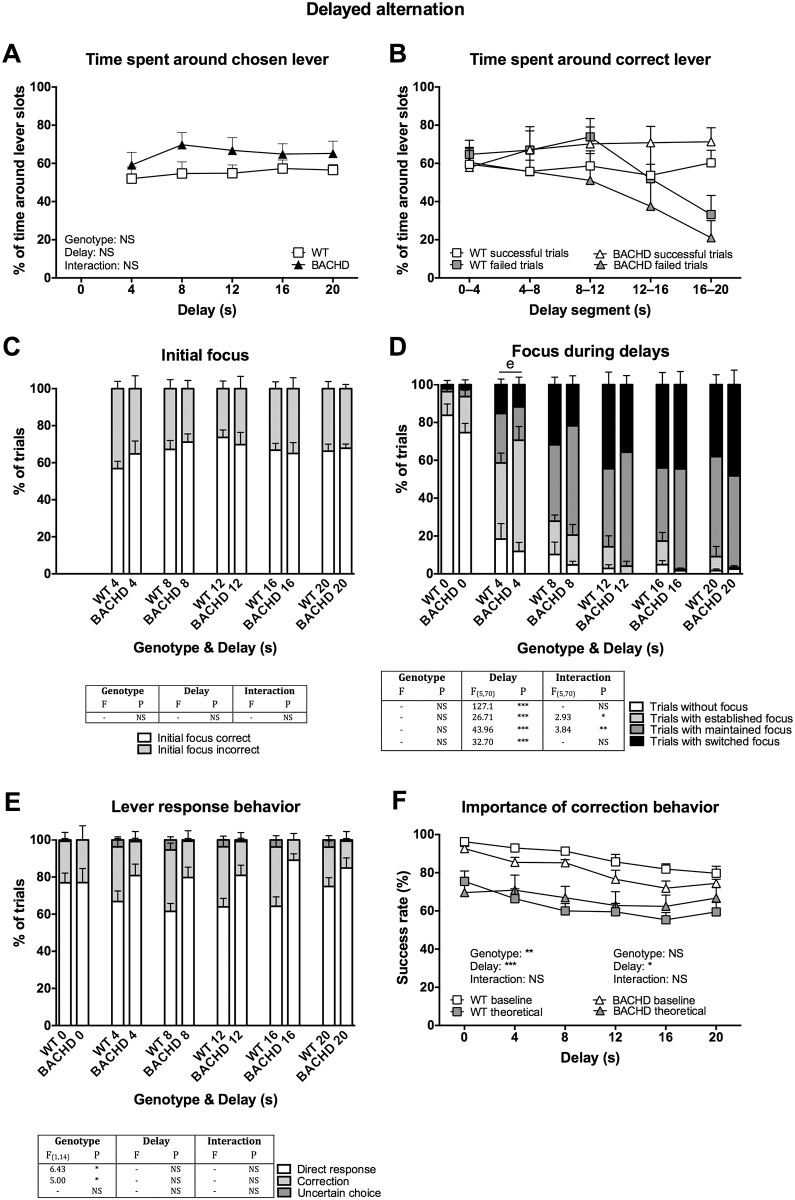
Video-scored behavior in relation to performance on delayed alternation. The graphs show various aspects of the behaviors scored from video recordings in relation to the rats' performance in the delayed alternation test. Graphs indicate group mean plus standard error. (B) concerns the 20-second delay step. In (F), the data that is labeled "theoretical" displays the theoretical success rates, as if the rats had responded according to their initial lever interest and not performed a correction behavior. Further details regarding the scored behaviors are described in the Material and Methods section. Results from two-way repeated measures ANOVA are shown inside the graphs. For (C)–(E), separate two-way ANOVAs were performed for each kind of behavior, and the respective results are indicated in small tables. Results from *post-hoc* analysis are indicated in case significant genotype differences were found. * (*P* < 0.05) ** (*P* < 0.01) *** (*P* < 0.001). In (D), ‘e’ notes a genotype difference (*P* < 0.05) for trials with established focus.

**Fig 11:**
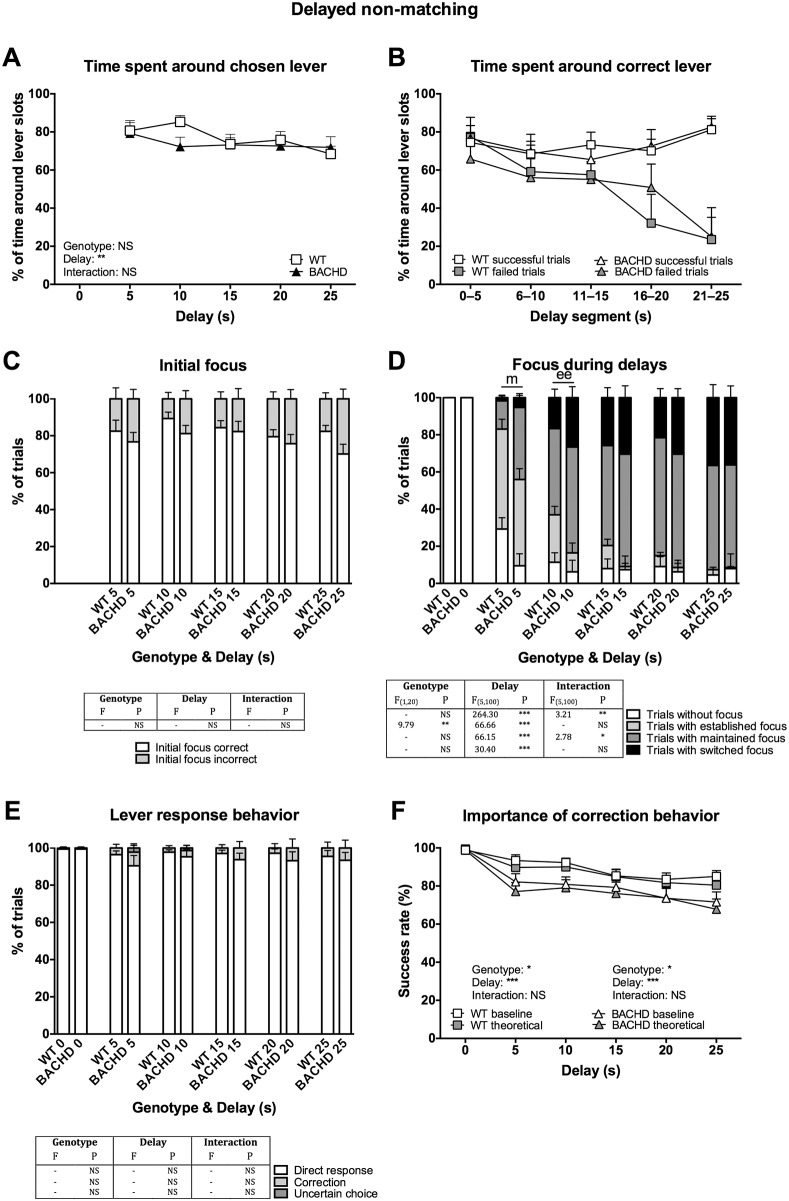
Video-scored behavior in relation to performance on delayed non-matching to position. The graphs show various aspects of the behaviors scored from video recordings in relation to the rats' performance in the delayed non-matching to position test. Graphs indicate group mean plus standard error. (B) concerns the 25-second delay step. In (F), the data that is labeled "theoretical" displays the theoretical success rates, as if the rats had responded according to their initial lever interest and not performed a correction behavior. Further details regarding the scored behaviors are described in the Material and Methods section. Results from two-way repeated measures ANOVA are shown inside the graphs. For (C)–(E), separate two-way ANOVAs were performed for each kind of behavior, and the respective results are indicated in small tables. Results from *post-hoc* analysis are indicated in case significant genotype differences were found. * (*P* < 0.05) ** (*P* < 0.01) *** (*P* < 0.001). In (D), ‘ee’ indicates a genotype difference (*P* < 0.01) for trials with established focus, and ‘m’ indicates a genotype difference (*P* < 0.05) for trials with maintained focus.

To further evaluate if WT and BACHD rats appeared to use different strategies when performing the tests, their body shifts and apparent focus towards a given side of the interactive wall were investigated in terms of their eventual lever choice. Rats in the delayed alternation test (Fig 10) showed a slight preference for making body shifts towards the lever they eventually responded to (Fig 10A). The preference remained stable with increasing delay, and although it appeared to be slightly stronger among BACHD rats, there were no significant genotype or genotype x delay interaction effects. In contrast, both WT and BACHD rats showed a strong preference for making body shifts towards the lever they eventually responded to during the non-matching to position test (Fig 11A). This preference dropped slightly with increasing delay duration (delay effect: F_(4,80)_ = 3.89, *P* < 0.01). Once again, there was no significant genotype or genotype x delay interaction effect. Additional analysis of the longest delay in each protocol was performed to evaluate the influence of the rats’ apparent lever focus on trial outcome. For both tests and both genotypes, correct lever choices were associated with maintaining a preference for the correct lever throughout the entire delay (Figs 10B and 11B). As above, this preference was notably stronger during the non-matching protocol compared to the alternation protocol. During trials with incorrect lever choices, the rats initially showed proper interest in the correct lever, but switched towards focusing on the incorrect lever during later phases of the delay. Once again, this behavior was apparent for rats of both genotypes and during both tests. It should be pointed out that proper statistics could not be performed for this analysis due to the limited amount of data available. As the noted behaviors appeared to constitute clear strategies for achieving high success rates on the two tests, further parameters were investigated to evaluate if the reduced success rate seen among BACHD rats might be explained by impaired strategy development and/or use. As noted, the rats’ initial focus during delays was frequently directed towards the correct lever. The frequency of trials with correct initial focus did not differ with delay duration or genotype for either test (Figs 10C and 11C) (data from trials with 0-second delays were excluded from the analysis due to the low number of trials with established focus). The frequencies of other focus-related behaviors were quantified as described in the Material and Methods section. During trials with 0-second delays, most rats did not establish a clear focus (Figs 10D and 11D). However, the frequency of this behavior dropped dramatically with delay duration (delay effect delayed alternation: F_(5,70)_ = 127.10, *P* < 0.001; delayed non-matching to position: F_(5,100)_ = 264.30, *P* < 0.001). The frequency of trials where a lever focus was only established (i.e. only one body shift motion was performed), was highest during trials with 4- and 5-second delays for the alternation and non-matching test, respectively. Like trials with no focus, their frequency clearly dropped with increasing delay duration (delay effect delayed alternation: F_(5,70)_ = 26.71, *P* < 0.001; delayed non-matching to position: F_(5,100)_ = 66.66, *P* < 0.001). In contrast, the frequency of trials with maintained or switched focus clearly increased with delay durations (delay effect of maintained focus delayed alternation: F_(5,70)_ = 43.96, *P* < 0.001; delay effect of maintained focus delayed non-matching to position: F_(5,100)_ = 66.15, *P* < 0.001; delay effect of switched focus delayed alternation: F_(5,70)_ = 32.70, *P* < 0.001; delay effect of switched focus delayed non-matching to position: F_(5,100)_ = 30.40, *P* < 0.001). There were trends indicating that WT rats showed a higher frequency of trials without focus compared to BACHD rats. This notion was supported by a significant genotype x interaction effect (F_(5,100)_ = 3.21, *P* < 0.01) for this parameter during the delayed non-matching test. Further, WT rats showed a lower frequency of trials with only established focus compared to BACHD rats, as indicated by significant genotype effects (delayed non-matching to position: F_(1,20)_ = 9.79, *P* < 0.01), genotype x delay interaction effects (delayed alternation: F_(5,70)_ = 2.93, *P* < 0.05), and post-test results (*P* < 0.05/0.01 on trials with 4- and 10-second delays for alternation and non-matching test, respectively). WT rats also showed a lower frequency of trials with maintained focus compared to BACHD rats, as indicated by significant genotype x delay interaction effects (delayed alternation: F_(5,70)_ = 3.84, *P* < 0.01; delayed non-matching to position: F_(5,100)_ = 2.78, *P* < 0.05) and significant *post-hoc* analysis results for trials with 5-second delays in the non-matching test (*P* < 0.05). There were no significant differences between genotypes in the frequency of trials with switched focus. It should be noted that although WT rats tended to show less trials with maintained focus, the ratio of maintained focus to switched focus trials did not differ between the genotypes (data not shown). Thus, the lower frequency of trials with maintained focus seen among WT rats in the analysis described above was likely a result of their lower tendency to perform body shifts (as indicated by the increased frequency of trials without focus).

The final behavioral aspect that was investigated concerned the rats’ behavior while performing the final lever response. In both tests, the most common behavior for both genotypes and all delays were direct responses (Figs 10E and 11E). Thus, rats rarely showed any interest in the non-chosen lever. However, during the delayed alternation test, there were still a considerable amount of corrections (Fig 10E). Notably, BACHD rats showed a higher frequency of direct responses compared to WT rats (genotype effect: F_(1,14)_ = 6.43, *P* < 0.05), while WT rats showed a higher frequency of corrections (genotype effect: F_(1,14)_ = 5.00, *P* < 0.05). The frequency of uncertain choices was marginally higher among WT rats, although it failed to reach statistical significance. As noted, the behaviors did not notably change with delay duration, although 0-second delay trials were the only ones where the aforementioned difference was not clearly present. The correction behavior still appeared to be important for the overall success rate of both WT and BACHD rats. Notably, the theoretical success rate of both genotypes (where the outcome of all correction trials had been adjusted to the hypothetical outcome of the response they initially intended to do) was markedly lower compared to their actual baseline (Fig 10F). While the actual baseline showed a similar performance deficit among BACHD rats as the one described above (genotype effect: F_(1,14)_ = 8.88, *P* < 0.01), there was no significant genotype difference in the theoretical data. Overall, these results were in clear contrast to the rats’ behavior during the delayed non-matching to position test (Fig 11E). During that test, rats of both genotypes rarely displayed corrections and uncertain responses, and no genotype differences were found for these behaviors. In line with this, the rats’ theoretical success rate did not clearly differ from their original baseline (Fig 11F). Thus, the BACHD rats’ performance deficit was apparent in both data sets (genotype effect, recorded data: F_(1,20)_ = 6.04, *P* < 0.05; genotype effect, theoretical data: F_(1,20)_ = 5.13, *P* < 0.05).

An overview of the main results found in the delayed alternation and delayed non-matching to position tests are show in Tables 1 and 2.

**Table 1:** Overview of results from the delayed alternation test.

Parameter	Results	Figure reference
Training needed to handle basic alternation task	BACHD rats required more training than WT rats during the first test age. The phenotype was less apparent but still present during the second test age and was fully resolved after further retesting.	Fig 3A
Training needed to progress through delay sets	BACHD rats required more training than WT rats during the first test age. The phenotype was less apparent but still present during the second and third test age and was fully resolved at the last test age.	S1 Fig
Overall success rate	BACHD rats showed a generally impaired performance with lower success rates compared to WT rats on all trial types.	Fig 4A, S2 Fig
Entries into pellet trough during delays	BACHD rats made fewer entries compared to WT rats. The phenotype was more pronounced on trials with long delays, although it did not appear to be due to BACHD rats gradually losing interest in the pellet trough with time.	Fig 5B and 5C
Time in pellet trough during delays	No overt difference was found between the genotypes in data recorded by the operant conditioning system, although a trend indicating that BACHD rats spent less time in the pellet trough compared to WT rats was found when manually scoring video recorded behaviors of the rats.	Figs 5D and 9A
Trial start latency	No difference was found between the genotypes during the first test age. During retesting, BACHD rats showed significantly longer trial start latencies compared to WT rats on trials with intermediate delay durations, which was found to be due to BACHD rats making frequent breaks for drinking.	S3A and S3C Fig
Lever response latency	No overt difference was found between the genotypes, although BACHD rats appeared to be slightly slower than WT rats during trials with 0-second delays.	S4A Fig
Pellet retrieval latency	No difference was found between the genotypes during the first two test ages. BACHD rats then appeared to become gradually slower with age, resulting in them being significantly slower than WT rats at the last test age.	S4B Fig
Omissions	No difference was found between the genotypes during the first test age. During retesting, BACHD rats showed significantly increased omission rates on trials with 0-, 4- and 8-second delays, which was found to be due to BACHD rats making frequent breaks for drinking.	S3B and S3D Fig
Video-scored behavior	During delays, rats of both genotypes were found to frequently exit the pellet trough and investigate the area around the retracted levers. There were discreet indications that this behavior functioned as a strategy for remembering which lever to respond to. There was no significant difference between the genotypes regarding this behavior, although BACHD rats tended to do it more frequently than WT rats. During lever responses, BACHD rats showed a reduced frequency of a type of correction behavior, and a corresponding increase in performing lever responses without hesitation, compared to WT rats. The correction behavior appeared to be important for maintaining a high success rate in the test.	Figs 9 and 10

**Table 2:** Overview of results from the delayed non-matching test.

Parameter	Brief description of phenotype	Figure reference
Training needed to handle basic non-matching task	No difference was found between the genotypes.	Fig 6A
Training needed to progress through delay sets	No difference was found between the genotypes.	S9 Fig
Overall success rate	BACHD rats showed unchanged success rate on trials without delays, while performance on trials with delays appeared to be generally impaired.	Fig 7A, S10 Fig
Trial start latency	No difference was found between the genotypes.	S12 Fig
Sample lever response latency	No difference was found between the genotypes.	S13A Fig
Food crib return latency	No difference was found between the genotypes.	Fig 8A
Entries into pellet trough during delays	BACHD rats made fewer entries compared to WT rats. The phenotype was more pronounced on trials with long delays, although it did not appear to be due to BACHD rats gradually losing interest in the pellet trough with time.	Fig 8B and 8C
Time in pellet trough during delays	No overt difference was found between the genotypes in data recorded by the operant conditioning system, although a trend indicated that BACHD rats spent less time in the pellet trough compared to WT rats. Manual video scoring of the behavior revealed a more pronounced phenotype of this nature.	Figs 8D and 9B
Latency to trigger choice step	No difference was found between the genotypes, although a trend indicated that BACHD rats were slightly slower than WT rats.	S11A Fig
Choice lever response latency	No difference was found between the genotypes.	S13B Fig
Pellet retrieval latency	BACHD rats were consistently slower than WT rats when retrieving the reward pellets.	S14A Fig
Omissions	BACHD rats showed a significantly higher number of trial start omissions, compared to WT rats	S11C Fig
Video-scored behavior	During delays, rats of both genotypes were found to frequently exit the pellet trough and investigate the area around the retracted levers. There were strong indications that this behavior functioned as a strategy for remembering which lever to respond to. BACHD rats performed this more frequently than WT rats. However, the video scoring did not reveal any behavioral differences that might explain the BACHD rats’ reduced success rate in the test.	Figs 9 and 11

## Discussion

### BACHD rats show no impairment when learning to perform simple instrumental response tasks

Our study did not reveal any overt differences between BACHD and WT rats during the initial habituation and lever training steps, with the exception of occasional indications that BACHD rats were slower at returning to the lever during CRF training. These findings are largely similar to what we have presented in previous publications [31], and what we have found in several studies that remain unpublished at this time. It should, however, be noted that in most of these studies the initial training steps were performed when the rats were 2–5 months old, and learning deficits might still be present in older animals. In line with this, it has been found that 18 months old BACHD rats (but not 2 and 8 months old rats) required more sessions than WT rats to reach criterion when learning to perform single nose pokes for food rewards [33]. However, no detailed analysis was performed to investigate if this phenotype was based on the rats having actual difficulties to associate the instrumental response with the delivery of a food pellet, or rather them being less interested in exploring the test chamber. Thus, while the BACHD rats’ ability to learn simple instrumental response tasks appears to be reliably intact at young ages, it is still unclear if it deteriorates with age.

### BACHD rats show slowed learning during alternation training, but not during non-matching to position training

Later training steps revealed clear differences between the alternation and the non-matching test. Specifically, while rats of both genotypes needed several training sessions before reaching criterion on the free alternation protocol, they required very little training to reach criterion on the free non-matching test. It is possible that this was due to the alternation protocol putting more strain on the rats’ inhibitory control. Essentially, it is reasonable to assume that rats have a strong tendency to return to a previously reinforced lever. During alternation protocols, this would cause them to have a high tendency to perform erroneous responses, and appropriate response inhibition would be required to achieve a high success rate. In contrast, the tendency to respond to the sample lever position during the non-matching protocols is likely low, as that response is never reinforced. Interestingly, BACHD rats showed a slowed learning compared to WT rats during the free alternation but not the free non-matching. This could have been due to them having specific problems with certain aspects of the alternation protocol (such as the suggested inhibitory control aspect), although it is also possible that the slowed learning represented a general learning deficit, which was not apparent during the non-matching test due to its relative simplicity. However, the latter hypothesis is unlikely to be true, as we have performed other complex cognitive tests without finding slowed learning among BACHD rats (unpublished results). In addition, the former hypothesis is to some extent supported by the BACHD rats’ generally impaired performance in the delayed alternation test (further discussed below). It should also be noted that the slowed learning among BACHD rats was likely not related to any underlying motivational deficits, as there were no differences in the number completed trials.

Despite the slowed learning among BACHD rats, the performance during criterion sessions was comparable between the genotypes during both the free alternation and free non-matching. The only exception was the longer pellet retrieval latencies seen among BACHD rats, which was present in both protocols. This phenotype has been found in almost all operant conditioning tests that have been run with the BACHD rats at our institute (seven longitudinal studies) and is described in previous publications [31]. Interestingly, similar phenotypes have been found in transgenic rats that carry a fragment of the HD-causing gene (TgHD rats) [34]. In the current study, the non-matching to position protocol offered additional information regarding this phenotype. Specifically, it allowed direct comparison between the pellet retrieval latency and the pellet trough return latency. These two parameters measured the latency to perform comparable motor behaviors, but aimed towards a pellet trough that either contained a reward pellet or was empty. While WT rats were clearly faster at moving to the pellet trough when there was a reward pellet present, BACHD rats showed comparable latencies in both situations. This could indicate a form of emotional blunting among BACHD rats. However, it is also possible that the BACHD rats were already moving at their maximum speed. More focused investigation of this phenotype is needed to better understand if its nature is motoric or psychological. Interestingly, TgHD rats have shown indications of emotional blunting in a sucrose solution consumption test [35].

### BACHD rats show reduced success rates in both the delayed alternation and delayed non-matching to position tests

The study’s main finding of interest was the consistently lower success rates found among BACHD rats in both the delayed alternation and non-matching to position test. The impairment seen in the delayed alternation test was not clearly affected by delay duration, suggesting that the rats had a general problem performing the basic task (i.e. alternation) rather than a short-term memory deficit. Similar phenotypes have been found in a knock-in HD mouse model [36] and rats with either striatal or prefrontal lesions [23–25]. The BACHD rats’ slowed learning during the free alternation training also supports the idea that they have problems handling the basic alternation task. It should, however, be noted that there were occasional tendencies indicating that the BACHD rats’ impairment became stronger with longer delay durations. Interestingly, such phenotypes have been found when lesioning specific fronto-striatal circuits [26], but also when lesioning the hippocampus [37]. Both pathologies could theoretically be present in the BACHD rats, as extensive protein aggregate formation has previously been found in the rats in these brain regions [30,38]. It should, however, also be noted that the apparent delay-dependent worsening of the phenotype might have been influenced by other aspects of the rats’ behavior (further discussed below). The impairment seen in the delayed non-matching to position test was similar to that of the delayed alternation test, although not identical. Longer delays did once again not appear to result in a stronger deficit, while the general presence of delays seemed to be crucial. Thus, the phenotype once again appeared to be due to the BACHD rats having general problems with the basic task, although it specifically concerned the delayed task. To our knowledge, similar phenotypes have not been reported elsewhere. Striatal lesions have been found to result in slight learning impairments in the delayed non-matching test [27], which were not seen in the current study. Further, fornix lesions have been found to result in delay-dependent impairments [39,40], while lesions to the prefrontal cortex and hippocampus have been found to produce a general drop in success rate [28,29]. The latter kind of phenotype was also found in a recent study of the performance of an HD knock-in mouse in a slightly different version of the test [41]. As noted though, these phenotypes do not appear to be directly comparable to the apparent biphasic curves found in the current study. As a final note, it is interesting that HD patients have shown general problems to perform accurately in a delayed pattern matching to sample test, although the impairment did not become more pronounced with increased delays [42].

### BACHD rats show other behavioral changes, although their influence on success rate is likely limited

Additional parameters were evaluated, as non-cognition based behavioral differences between BACHD and WT rats could have influenced the success rate in the two tests, and needed to be considered. The results revealed that there were indeed several behavioral differences between BACHD and WT rats, although few appeared to be directly related to the rats’ success rates. During the delayed alternation test, BACHD rats were found to be slower at performing the first head entry of the delay, performed fewer head entries during the delays and showed longer trial start latencies for trials with intermediate delay durations. Out of these parameters, the difference in trial start latencies was the only factor that appeared to be related to failed trials. Still, BACHD and WT rats showed comparable trial start latencies during the first test age, while BACHD rats already presented an overall lower success rate. Similarly, when only trials without water consumption were considered, there was no difference between genotypes in terms of trial start latencies, while the reduced success rate among BACHD rats was still present. Thus, although the slowed trial start latency among BACHD rats most likely affected their performance to some extent, it was unlikely the main cause of their reduced success rate. There were fewer differences between the BACHD and WT rats’ behavior during the delayed non-matching test. Most notably, BACHD rats again performed fewer head entries than WT rats during delays. However, detailed analysis suggested once again that this difference was unlikely to explain the difference in success rate. As the reduced number of head entries was still consistently found in the two tests, it is worth noting that similar phenotypes have been found in rats with striatal lesions [24], although not consistently [23]. Ultimately, despite the various noted behavioral differences found between BACHD and WT rats it is likely that underlying cognitive changes were the main cause of their reduced success rates. However, additional non cognition-related behavioral differences likely still influenced the overall appearance of the phenotype to some extent. When considering this, it is also noteworthy that some parameters appeared to only affect certain trial types. Specifically, the relationship between trial start latencies and trial outcome in the delayed alternation test appeared to primarily concern trials with short delays, and was restricted to BACHD rats. In contrast, the WT rats’ success on trials with short delays appeared to be more related to their lever response latencies. Specific parameters also appeared to be related to failure on trials with short delays during the delayed non-matching test (i.e. trial start latency, pellet trough return latency, latency to trigger choice step and choice lever response latency). These aspects complicate the task of assessing the appearance of the rats’ actual cognitive impairment, making it possible that the current interpretations (i.e. cognitive impairments resulting in BACHD rats showing an overall impaired performance in the delayed alternation test, and a biphasic impairment in the delayed non-matching to position test) are not entirely true. Specifically, the BACHD rats’ lower success rate on trials with 0-second delays during the delayed alternation test might have been related to non-cognitive behaviors influencing their trial start latencies. Thus, their true cognitive impairment might have been more comparable to the one seen in the delayed non-matching to position test, which would also be more in line with their unchanged success rate during the free alternation protocols.

Although the difference in the number of head entries performed during delays did not appear to be connected to the reduced success rate among BACHD rats, a difference in delay behavior might still indicate altered motivation, attention or strategy. Thus, additional analysis of this phenotype was of interest. The phenotypes were first validated with video scoring that indicated that BACHD rats indeed spent less time than WT rats being in an arguably central position during delays (although the phenotype was quite discreet during the delayed alternation test). Certain aspects argued against the phenotype being caused by motivational differences. First, the reduced number of entries was present on each segment of the longer delay steps, suggesting that the phenotype was not due to the BACHD rats simply losing interest as time passed. In addition, the number of entries was not strongly affected by changing the food restriction protocol of WT rats. BACHD rats were, however, found to frequently turn around to drink water during delays in the delayed alternation test, suggesting a change in their relative interest in food and water. It is worth noting that this behavior appeared to be the main cause of the BACHD rats’ peculiar pattern of trial start latencies and omissions during the test. Further, although the trial start latencies themselves appeared to have only limited influence on the success rate (see above), it is still possible that the drinking behavior had caused the trends indicating that the BACHD rats’ performance deficit worsened with longer delays. Regardless, although the drinking behavior might have affected the BACHD rats’ success rate, and definitely contributed to their lower number of head entries performed during delays, both phenotypes were still present when trials with drinking were excluded.

### BACHD and WT rats develop comparable strategies to maintain high success rates on the two tests

As noted, it was subsequently found that BACHD rats had a higher frequency of leaving the pellet trough to investigate the area around it. This could indicate a change in attention and strategy, although there were no indications of either one in the current study. Both BACHD and WT rats preferentially made body shifts towards the lever they would eventually respond to, and appeared to focus on the lever that would give a correct response. Thus, although BACHD rats made these movements at a higher frequency than WT rats, their use and relevance to lever choices did not notably differ between the genotypes on either test. The increased use of body shifts seen among BACHD rats might, however, indicate that they were more dependent on the strategy than WT rats. This would be in line with the trend suggesting that BACHD rats had a slightly stronger preference for making body shifts towards the chosen lever during the delayed alternation test. This idea should be further evaluated by comparing performance in setups or protocols that make the use of this strategy more difficult, such as placing the levers and pellet trough on opposite walls, placing walls between the wall sections containing the lever slots and the pellet trough, or adding limits so that trials are cancelled if the rats exit the pellet trough. Still, the importance of the body shifts for accurate performance during the delayed alternation test is somewhat uncertain, as the rats did not show a particularly strong focus for the selected lever. Thus, although the rats clearly used the body shifts as part of a strategy during the delayed non-matching test, thee reason for at all performing them (and the consistent finding of BACHD rats performing them more frequently than WT rats) might be due to more general and not strategy-related behaviors. Interestingly, transgenic rats carrying a fragment of the HD-causing gene have been found to show a high frequency of early withdrawals from nose poke modules during a choice reaction time test, which was suggested to be due to impaired response inhibition [43]. This phenotype is arguably similar to the one found in the current study.

Strategies similar to the body shifts described here have been found in other studies of rats performing the delayed non-matching to position test [40]. That particular study also indicated that fronto-striatal lesions, which resulted in reduced success rate, also affected these mediating behaviors. Among other things, lesioned rats showed an increased frequency of changing focus from one lever to another during delays. Due to this, we investigated similar parameters in the current study. In both tests and genotypes, there were indications that maintaining focus on the correct lever throughout the delay was related to a successful outcome, while switching focus to the wrong lever was related to failed trials. However, there were no clear indications that BACHD rats switched focus more frequently than WT rats. In addition, there were no differences regarding how often the rats’ initial focus was on the correct lever. It should, however, be noted that the scoring method used here (i.e. a rats’ apparent focus being based on the first and last body shift) was limited. However, more elaborate scoring (such as judging the rat’s apparent focus based on the percentage of time spent around a given lever) would have suffered from similar limitations due to the low number of body shifts that were performed (roughly four for WT and six for BACHD rats during the longest delay). Further insight into the rats’ focus-shifting behavior might still be gained through the analysis of more data, using a more elaborate scoring protocol, but it is beyond the scope of the current study.

### BACHD rats show a reduced frequency of correction behaviors during delayed alternation performance

The rats’ behavior while performing lever pushes was also investigated. This scoring indicated that most rats responded without hesitation during the choice step of the delayed non-matching test. This was most likely due to a strong association between their body shift and the planned lever response. Thus, the main decision regarding which lever to push was likely made already during the delay step. This might also explain why both WT and BACHD rats were faster at responding to choice levers than to sample levers. All in all, these aspects question to what extent the test really evaluated short-term memory rather than the rats’ ability to establish and maintain focus on the correct lever. Direct responses also constituted the majority of responses made in the delayed alternation test. However, there was a considerable frequency of correction behaviors, where the rats would first start moving towards one lever but change their mind and respond to the other one. The importance of this behavior was indicated by the dramatically lower success rate found when the rats’ theoretical performance was considered (i.e. success rate as if they had responded according to their initial lever choice). Importantly, the frequency of correction behaviors was higher among WT rats than BACHD rats. Further, there was no difference between WT and BACHD rats in their theoretical success rates. Thus, it is likely that the reduced frequency of correction behaviors among BACHD rats was connected to their lower success rate in the delayed alternation test. Still, in connection to the discussions above, it is noteworthy that there was no clear difference in the frequency of corrections during trials with 0-second delays. Regardless, the reduced frequency of correction behavior might be an indication that BACHD rats have difficulties inhibiting already initiated responses. This would suggest an impairment regarding a quite specific aspect of response inhibition, which should be further investigated in tests that probe this [44–46]. Evaluating the BACHD rats’ performance in such tests might also help to determine if the impairment in the delayed alternation test truly concerned a failure to inhibit erroneous responses, as opposed to a failure to realize that the initiated responses would be erroneous. Interestingly, changes in neuronal signaling have been found in HD patients during performance of tests where they had to inhibit ongoing motor responses [47]. In addition, HD patients [48], HD mouse models [49] and BACHD rats [50] have all been found to show impaired performance in other tests of response inhibition. It should, however, be noted that the study performed on BACHD rats did not conclusively show that the response inhibition impairment concerned a baseline deficit rather than a response to a change in protocol.

### The noted phenotypes generally remained stable with increasing age

As noted, the phenotypes found in the two tests did not appear to change with age. Due to the progressive nature of HD, one would typically expect that disease-related phenotypes in animal models would worsen when they grow older. Indeed, other phenotypes found in the BACHD rats have been shown to progressively worsen, already while the rats were a few months old [30]. However, the neuropathology of the BACHD rats has not been fully elucidated yet. Although loss of dopamine 2 receptors has been implicated in old animals, and although there is a gradual accumulation of huntingtin aggregates with age [30], it is not clear if this results in progressive loss of function in fronto-striatal circuits. The current results would suggest that it does not. Thus, the impairments found here might be due to neuropathology caused by the general presence of mutant huntingtin, rather than its progressive accumulation. Alternatively, the impairments might be due to neuropathology caused by developmental deficits. Specifically, male BACHD rats have been found to be smaller than their WT littermates [31] and consistently have smaller brains (unpublished results). At this point, it is unclear if this developmental deficit only regards size or also functionality. Finally, it should be considered that the rats in the current study spent roughly half of their life actively being assessed in the respective tests. This frequent behavioral evaluation might have acted as environmental enrichment, and might have counteracted any progression that would have occurred if less frequent training were used. To evaluate this further, additional tests should be run where test ages are spaced further apart or performed with separate test groups.

### Conclusions and final remarks

BACHD rats showed impaired performance in both the delayed alternation and delayed non-matching test in the current study. The phenotypes were already present at 2–4 months of age and did not appear to progressively worsen with age. In both tests, the rats appeared to primarily have problems handling the basic task, while short-term memory remained intact. The impairment found in the delayed alternation test seemed to in part be caused by a failure to correct ongoing erroneous responses, which in turn could be due to deficits in attention and/or inhibitory control. It is currently unclear what specific behavioral differences caused the impaired performance in the delayed non-matching to position test, although it is likely related to the distinct mediating behaviors that both WT and BACHD rats used. Importantly, arguably similar performance deficits have been found in other HD models and rats with fronto-striatal lesions, suggesting that the BACHD rats’ phenotypes are caused by HD-related neuropathology. In addition, the phenotypes were not affected by a change in motivation and hunger, suggesting that the impairments likely reflect true cognitive deficits rather than artifacts due to motivational differences between WT and BACHD rats.

As a side note, using water bottles during operant conditioning tests might not be optimal when working with BACHD rats. During delayed alternation training, the BACHD rats took frequent breaks to consume water, which dramatically affected their trial start latencies and omission rates. It is currently not clear why the rats developed this behavior, as it has not been found in other operant condition tasks performed at our institute. In addition, extensive control tests were run with the delayed alternation rats to investigate their thirst response to being fed reward pellets in various conditions. However, there were no indications that BACHD rats became thirstier than WT rats when consuming reward pellets.

## Supporting Information

S1 Fig Sessions required to progress through delayed alternation training. The graphs show the total number of sessions required for progressing through the series of delayed alternation protocols at the different test ages, with gradually increasing delay durations that were implemented before the training on the final delay set had started. The values were adjusted for the change in criterion that was made after the first test age. Rats, which did not reach criterion on each protocol, were excluded from the analysis. Plots indicate single values for individual rats. Note that the scale on the y-axis differs between the graphs. Results from *t*-test or Mann-Whitney U test are indicated in case significant genotype differences were present. * (*P* < 0.05) ** (*P* < 0.01) *** (*P* < 0.001).
(TIFF)

S2 Fig Success rate per delay in the delayed alternation test during retesting. The graphs show the success rate on trial types with delays of different durations in the delayed alternation test. Each graph shows the stable baseline performance of rats maintained on the standard food restriction protocol. Curves display group mean plus standard error. Results from two-way repeated measures ANOVA are shown inside the graphs. For (A), results from *post-hoc* analysis are indicated in case significant genotype differences were found. * (*P* < 0.05) ** (*P* < 0.01) *** (*P* < 0.001).
(TIFF)

S3 Fig Trial start latency and omissions during delayed alternation. The graphs show trial start latency and omissions during the delayed alternation protocol. (A) and (B) show the behavior at the four-month test age, while (C) and (D) show the mean performance at the three older ages. Graphs indicate group mean plus standard error. Results from two-way repeated measures ANOVA are shown inside the graphs. Results from *post-hoc* analysis are indicated in case significant genotype differences were found. * (*P* < 0.05) ** (*P* < 0.01) *** (*P* < 0.001).
(TIFF)

S4 Fig Additional parameters of delayed alternation performance. The graphs show the last two parameters investigated for delayed alternation performance. (A) is based on the overall performance on all test ages, as no significant change with age was found for the parameter. Graphs indicate group mean plus standard error. Results from two-way repeated measures ANOVA are shown inside the graphs. Results from *post-hoc* analysis are indicated in case significant genotype differences were found. * (*P* < 0.05) ** (*P* < 0.01) *** (*P* < 0.001).
(TIFF)

S5 Fig Parameters indicating success or failure on delayed alternation. The graphs show some of the parameters of the delayed alternation protocol with performance of WT and BACHD rats separated for successful and failed trials. All graphs were constructed based on the mean performance over all test ages, as the relation to trial outcome did not noticeably change with age. Graphs indicate group mean plus standard error. Results from two-way repeated measures ANOVA are shown inside the graphs. Results from *post-hoc* analysis are indicated in case significant genotype differences were found. * (*P* < 0.05) ** (*P* < 0.01) *** (*P* < 0.001).
(TIFF)

S6 Fig Effect of food restriction adjustment on success rate in delayed alternation test. The graphs show the WT rats' performance in the delayed alternation test during two different food restriction settings at the four investigated ages. Graphs indicate group mean plus standard error. Results from two-way repeated measures ANOVA are shown inside the graphs. Results from *post-hoc* analysis are indicated in case significant genotype differences were found. * (*P* < 0.05) ** (*P* < 0.01) *** (*P* < 0.001).
(TIFF)

S7 Fig Effect of food restriction adjustment and extended training on delayed alternation parameters. The graphs show some of the parameters of the delayed alternation protocol, comparing performance of WT and BACHD rats during their initial baseline with performance after changing food restriction protocol or given extended training, respectively. All graphs were constructed based on the mean performance over all test ages, as the effect of changing food restriction protocol or giving extended training did not noticeably change with age. Graphs indicate group mean plus standard error. Results from two-way repeated measures ANOVA are shown inside the graphs. Results from *post-hoc* analysis are indicated in case significant differences between baselines were found. * (*P* < 0.05) ** (*P* < 0.01) *** (*P* < 0.001).
(TIFF)

S8 Fig Effect of food restriction adjustment on omissions during delayed alternation. The graphs show the effect of food restriction adjustment and extended training on the number of trial start omissions performed during the delayed alternation test. All graphs were constructed based on the mean performance over all test ages, as the effect of changing food restriction protocol or giving extended training did not noticeably change with age. Graphs indicate group mean plus standard error. Results from two-way repeated measures ANOVA are shown inside the graphs. Results from *post-hoc* analysis are indicated in case significant differences between baselines were found. * (*P* < 0.05) ** (*P* < 0.01) *** (*P* < 0.001).
(TIFF)

S9 Fig Sessions required to progress through delayed non-matching to position training. The graphs show the total number of sessions required for progressing through the series of delayed non-matching to position protocols with gradually increasing delay durations, which were implemented before the training on the final delay set had started. The values were adjusted for the change in criterion that was made after the first test age. Rats that did not reach criterion on each protocol were excluded from the analysis. Plots indicate single values for individual rats. Note that the scale on the y-axis differs between (A) and the remaining graphs. Results from *t*-test or Mann-Whitney U test are indicated in case significant genotype differences were present. * (*P* < 0.05) ** (*P* < 0.01) *** (*P* < 0.001).
(TIFF)

S10 Fig Success rate per delay in the delayed non-matching to position test during retesting. The graphs show the age development of success rate on trial types with delays of different durations in the delayed non-matching test. Each graph shows the stable performance found when rats were maintained on the standard food restriction protocol. Curves display group mean plus standard error. Results from two-way repeated measures ANOVA are shown inside the graphs. Results from *post-hoc* analysis are indicated in case significant genotype differences were found. * (*P* < 0.05) ** (*P* < 0.01) *** (*P* < 0.001).
(TIFF)

S11 Fig Latency to trigger choice step and omissions for delayed non-matching to position. The graphs show the latency to initiate the choice step, related omissions and omissions overview during the delayed non-matching to position protocol. Graphs display the mean performance over all ages, as no significant differences in the rats’ behavior at different ages was found. (A) and (B) indicate group mean plus standard error. (C) indicates the performance of individual rats. For (A) and (B), results from two-way repeated measures ANOVA are shown inside the graphs, and results from *post-hoc* analysis are indicated in case significant genotype differences were found. For (C), results from *t*-test or Mann-Whitney U test are indicated in case the genotypes differed significantly. * (*P* < 0.05) ** (*P* < 0.01) *** (*P* < 0.001).
(TIFF)

S12 Fig Trial start latency in the delayed non-matching to position test. The graph shows the latency to initiate trials on the different test ages of the delayed non-matching to position protocol. The curve indicates group mean plus standard error. Results from two-way repeated measures ANOVA are shown inside the graph, and results from *post-hoc* analysis are indicated in case significant genotype differences were found. * (*P* < 0.05) ** (*P* < 0.01) *** (*P* < 0.001).
(TIFF)

S13 Fig Lever response latencies during delayed non-matching to position. The graphs show the latencies to respond to a lever during either the sample step or the choice step of the delayed non-matching to position protocol. (A) and (B) display the comparison between WT and BACHD for both response latencies, while (C) and (D) display comparisons between the type of response latencies for both genotypes. Graphs display mean performance over all ages, as no significant differences in the rats’ behavior at different ages was found. Curves indicate group mean plus standard error. Results from two-way repeated measures ANOVA are shown inside the graphs, and results from *post-hoc* analysis are indicated for data points where significant genotype differences were found. * (p < 0.05) ** (p < 0.01) *** (p < 0.001).
(TIFF)

S14 Fig Reward pellet retrieval latency during delayed non-matching to position. (A) shows the mean pellet retrieval latency of WT and BACHD rats during the delayed non-matching to position protocol at all investigated ages. (B) shows a comparison of the mean pellet retrieval latency with the mean latency to return to the pellet trough after pushing the sample lever. For this, the mean of all investigated ages and trial types were used, as the phenotypes or differences between latencies did not clearly change with age. Curves indicate group mean plus standard error. Results from two-way repeated measures ANOVA are shown inside the graphs, and results from *post-hoc* analysis are indicated in case significant genotype differences were found. * (*P* < 0.05) ** (*P* < 0.01) *** (*P* < 0.001).
(TIFF)

S15 Fig Parameters indicating success or failure for WT rats in the delayed non-matching to position test. The graphs show some of the parameters of the delayed non-matching to position protocol performance of WT rats separated for successful and failed trials. All graphs were constructed using the mean performance over all ages, as the parameters’ relation to trial outcome did not noticeably change between test ages. In addition, this was necessary to obtain data for failed 0-second delay trials for all rats. Curves indicate group mean plus standard error. Results from two-way repeated measures ANOVA are shown inside the graphs, and results from *post-hoc* analysis are indicated in case significant genotype differences were found. * (*P* < 0.05) ** (*P* < 0.01) *** (*P* < 0.001).
(TIFF)

S16 Fig Parameters indicating success or failure for BACHD rats in the delayed non-matching to position test. The graphs show some of the parameters of the delayed non-matching to position protocol performance of BACHD rats separated for successful and failed trials. All graphs were constructed using the mean performance over all ages, as the parameters’ relation to trial outcome did not noticeably change between test ages. In addition, this was necessary to obtain data for failed 0-second delay trials for all rats. Curves indicate group mean plus standard error. Results from two-way repeated measures ANOVA are shown inside the graphs, and results from *post-hoc* analysis are indicated in case significant genotype differences were found. * (*P* < 0.05) ** (*P* < 0.01) *** (*P* < 0.001).
(TIFF)

S17 Fig Effect of food restriction adjustment on success rate in delayed non-matching to position. The graphs show the WT rats' performance in the delayed non-matching to position test during two different food restriction settings at the four investigated age. Graphs indicate group mean plus standard error. Results from two-way repeated measures ANOVA are shown inside the graphs. Results from *post-hoc* analysis are indicated in case significant genotype differences were found. * (*P* < 0.05) ** (*P* < 0.01) *** (*P* < 0.001).
(TIFF)

S18 Fig Effect of food restriction adjustment of WT rats in the delayed non-matching to position test. The graphs show some of the parameters of the delayed non-matching to position protocol performance of WT rats separated for standard and alternative food restriction protocols. All graphs were constructed using the mean performance over all ages, as the parameters’ relation to motivational state did not noticeably change between test ages. Curves indicate group mean plus standard error. Results from two-way repeated measures ANOVA are shown inside the graphs, and results from *post-hoc* analysis are indicated in case significant genotype differences were found. * (*P* < 0.05) ** (*P* < 0.01) *** (*P* < 0.001).
(TIFF)

S19 Fig Effect of extended training of BACHD rats in the delayed non-matching to position test. The graphs show some of the parameters of the delayed non-matching to position protocol performance of BACHD rats separated for the baselines after initial and extended training. All graphs were constructed using the mean performance over all ages, as the parameters’ relation to the amount of training did not noticeably change between test ages. Curves indicate group mean plus standard error. Results from two-way repeated measures ANOVA are shown inside the graphs, and results from post-hoc analysis are indicated in case significant genotype differences were found. * (*P* < 0.05) ** (*P* < 0.01) *** (*P* < 0.001).
(TIFF)

S20 Fig Trial start omissions during different baselines of delayed non-matching to position. The graph shows the number of omissions during the initial baselines and after either a change in food restriction protocol or extended training on the delayed non-matching to position protocol. The graph was constructed using the mean performance over all ages, as the parameters’ relation to food restriction or extended training did not noticeably change between test ages. The curve indicates group mean plus standard error. Results from two-way repeated measures ANOVA are shown inside the graph, and results from *post-hoc* analysis are indicated in case significant genotype differences were found. * (*P* < 0.05) ** (*P* < 0.01) *** (*P* < 0.001).
(TIFF)
