# Supplementary figures and images for: The BACHD Rat Model of Huntington Disease Shows Signs of Fronto-Striatal Dysfunction in Two Operant Conditioning Tests of Short-Term Memory
Source: PLoS One. 2017 Jan 3;12(1):e0169051. doi: 10.1371/journal.pone.0169051 (PMC5207398; doi:10.1371/journal.pone.0169051)

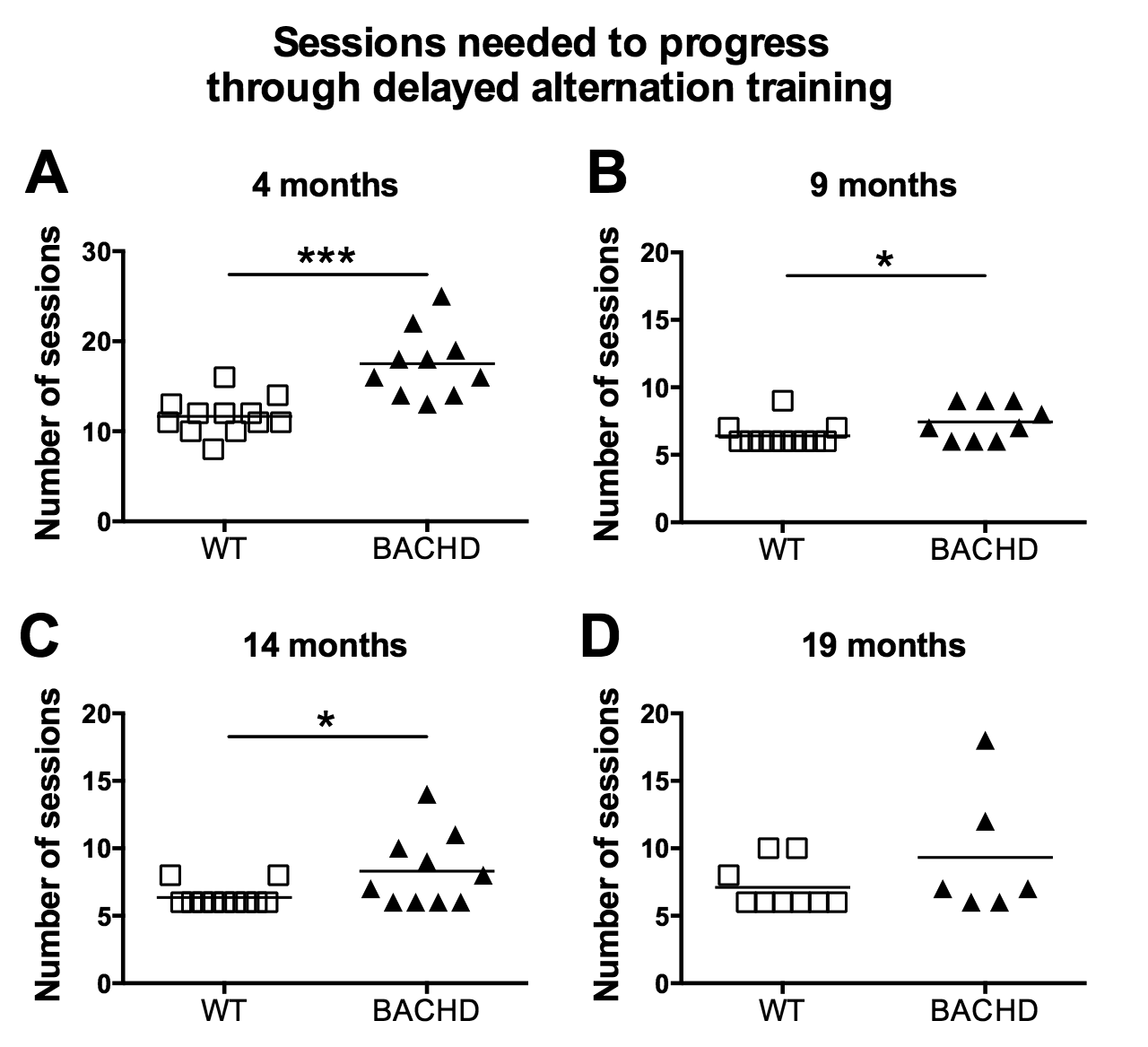

Supplement: S1 Fig — The graphs show the total number of sessions required for progressing through the series of delayed alternation protocols at the different test ages, with gradually increasing delay durations that were implemented before the training on the final delay set had started. The values were adjusted for the change in criterion that was made after the first test age. Rats, which did not reach criterion on each protocol, were excluded from the analysis. Plots indicate single values for individual rats. Note that the scale on the y-axis differs between the graphs. Results from t-test or Mann-Whitney U test are indicated in case significant genotype differences were present. * (P < 0.05) ** (P < 0.01) *** (P < 0.001). (TIFF) [file pone.0169051.s001.tiff]

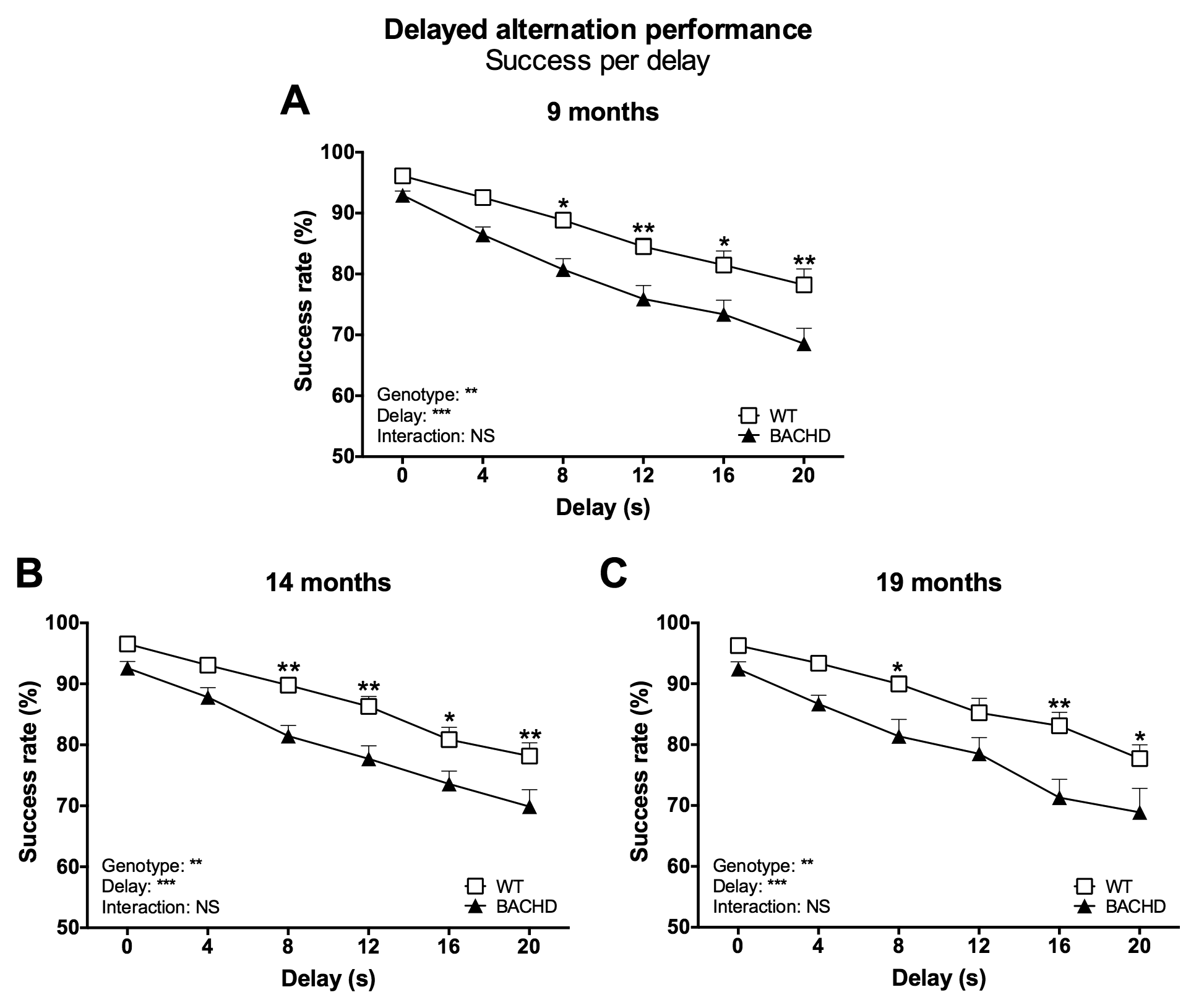

Supplement: S2 Fig — The graphs show the success rate on trial types with delays of different durations in the delayed alternation test. Each graph shows the stable baseline performance of rats maintained on the standard food restriction protocol. Curves display group mean plus standard error. Results from two-way repeated measures ANOVA are shown inside the graphs. For (A), results from post-hoc analysis are indicated in case significant genotype differences were found. * (P < 0.05) ** (P < 0.01) *** (P < 0.001). (TIFF) [file pone.0169051.s002.tiff]

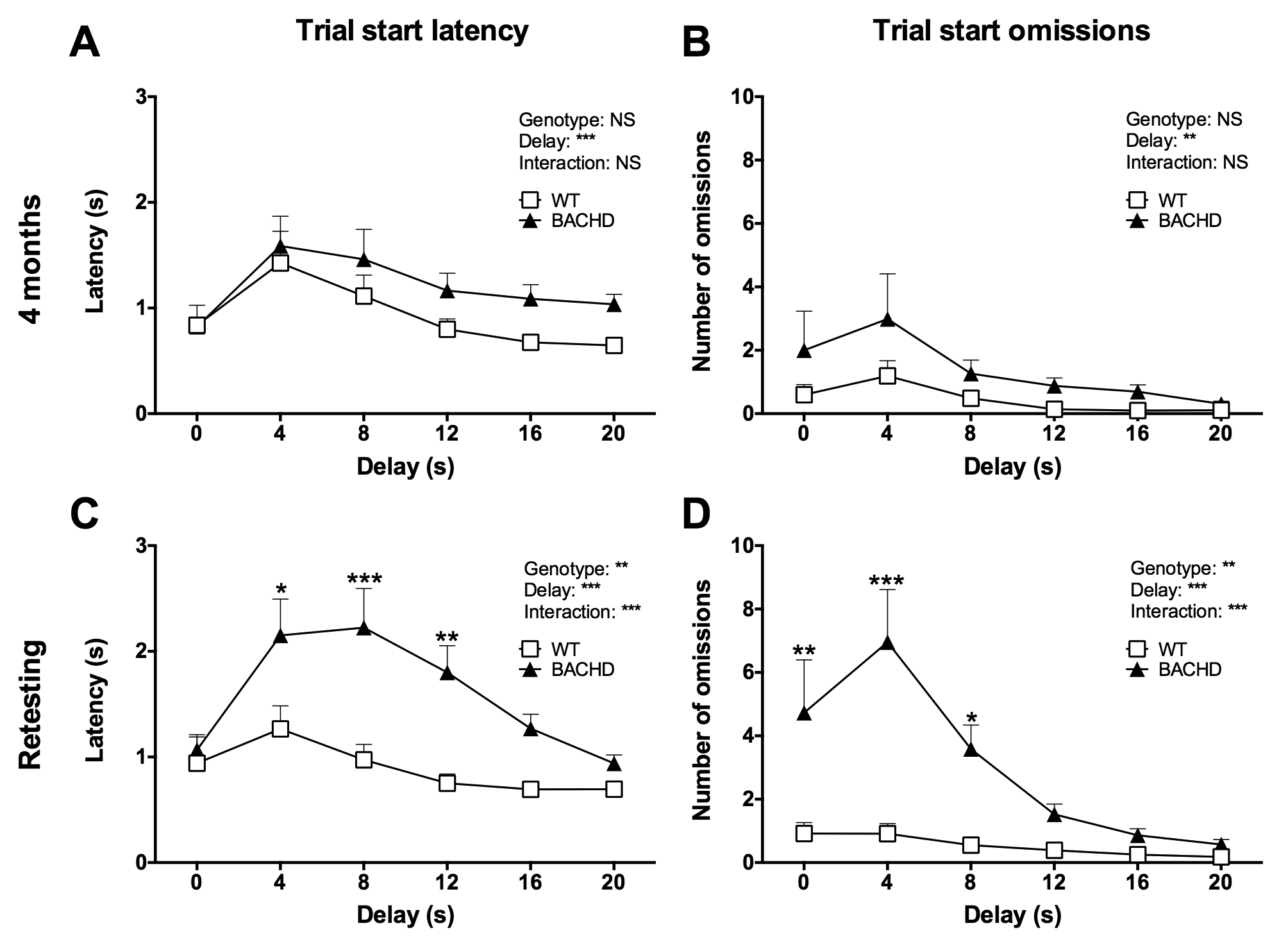

Supplement: S3 Fig — The graphs show trial start latency and omissions during the delayed alternation protocol. (A) and (B) show the behavior at the four-month test age, while (C) and (D) show the mean performance at the three older ages. Graphs indicate group mean plus standard error. Results from two-way repeated measures ANOVA are shown inside the graphs. Results from post-hoc analysis are indicated in case significant genotype differences were found. * (P < 0.05) ** (P < 0.01) *** (P < 0.001). (TIFF) [file pone.0169051.s003.tiff]

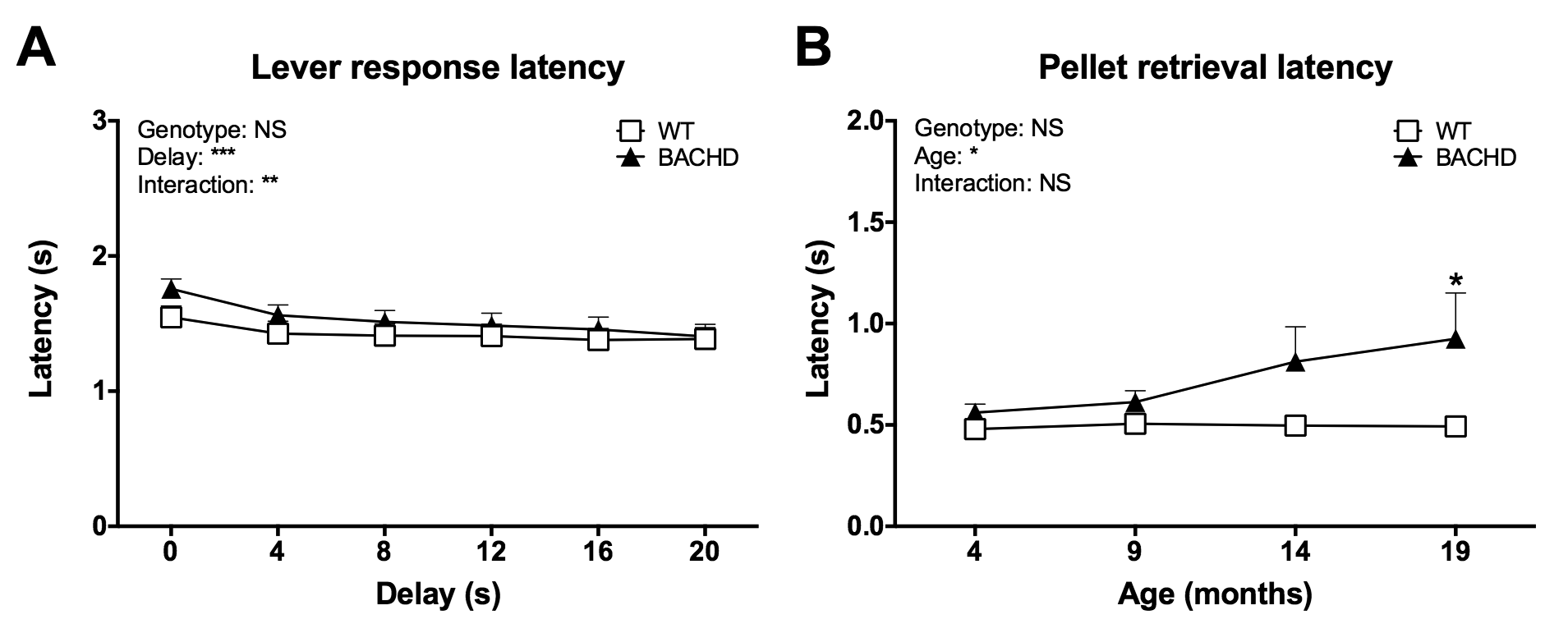

Supplement: S4 Fig — The graphs show the last two parameters investigated for delayed alternation performance. (A) is based on the overall performance on all test ages, as no significant change with age was found for the parameter. Graphs indicate group mean plus standard error. Results from two-way repeated measures ANOVA are shown inside the graphs. Results from post-hoc analysis are indicated in case significant genotype differences were found. * (P < 0.05) ** (P < 0.01) *** (P < 0.001). (TIFF) [file pone.0169051.s004.tiff]

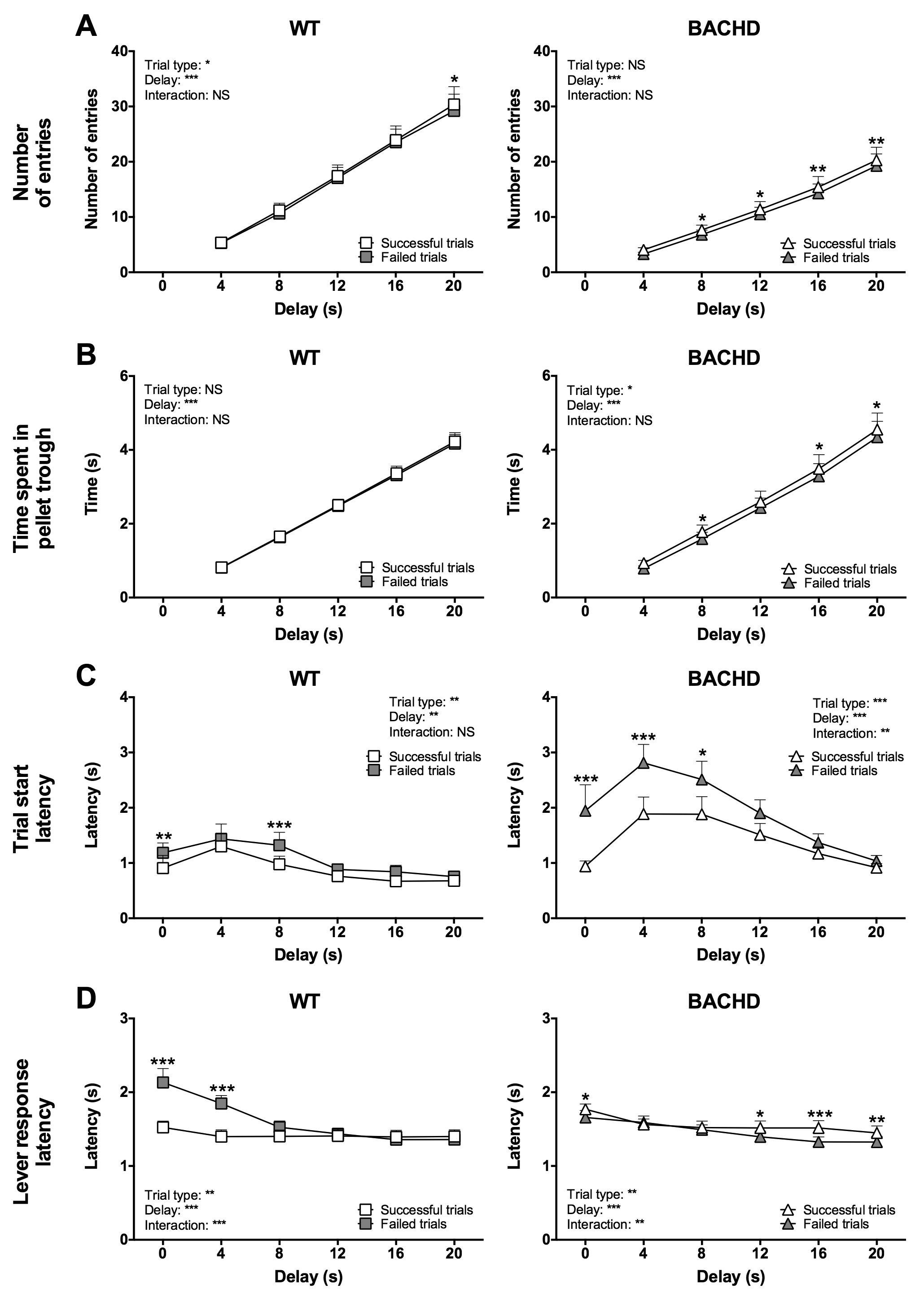

Supplement: S5 Fig — The graphs show some of the parameters of the delayed alternation protocol with performance of WT and BACHD rats separated for successful and failed trials. All graphs were constructed based on the mean performance over all test ages, as the relation to trial outcome did not noticeably change with age. Graphs indicate group mean plus standard error. Results from two-way repeated measures ANOVA are shown inside the graphs. Results from post-hoc analysis are indicated in case significant genotype differences were found. * (P < 0.05) ** (P < 0.01) *** (P < 0.001). (TIFF) [file pone.0169051.s005.tiff]

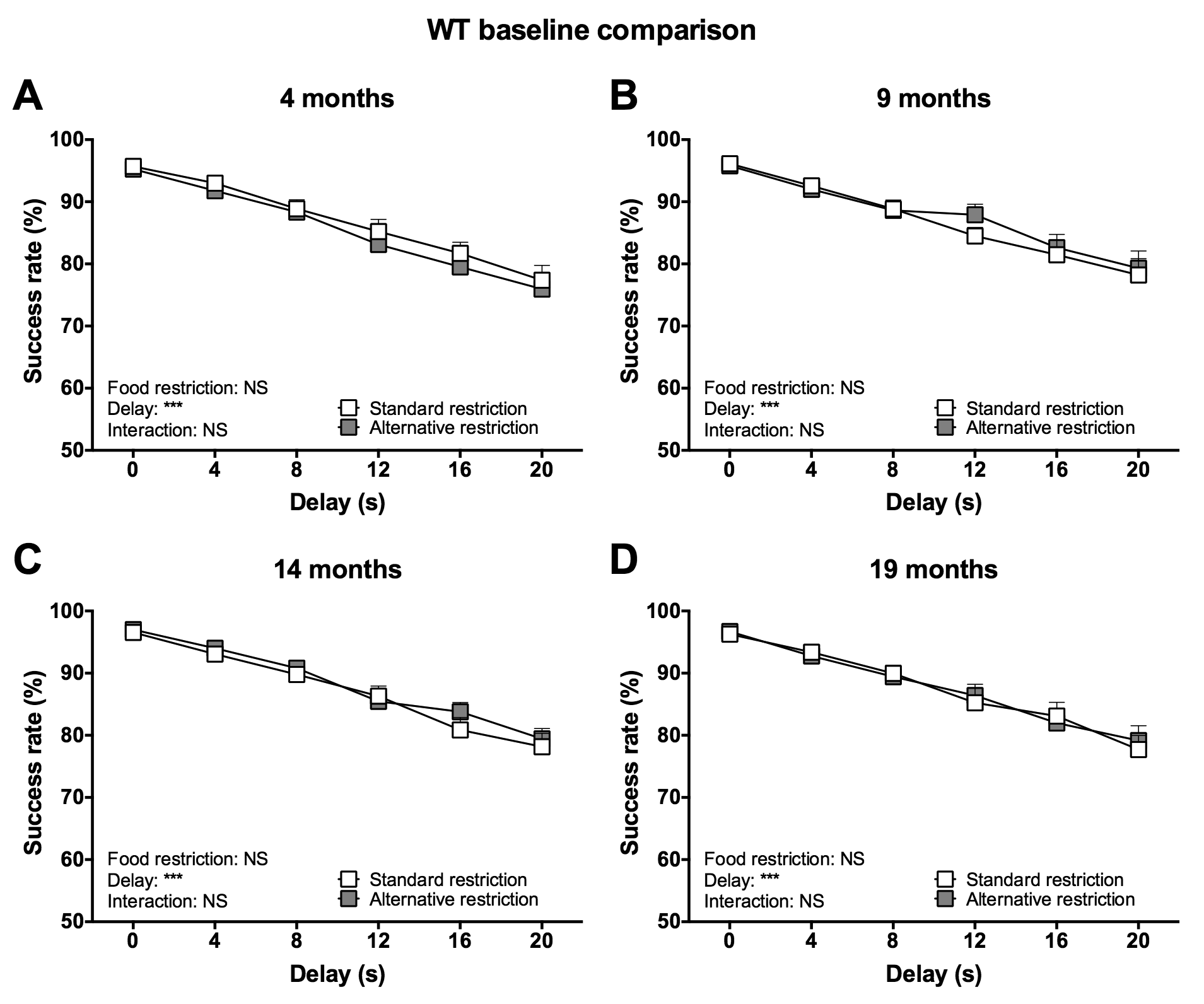

Supplement: S6 Fig — The graphs show the WT rats' performance in the delayed alternation test during two different food restriction settings at the four investigated ages. Graphs indicate group mean plus standard error. Results from two-way repeated measures ANOVA are shown inside the graphs. Results from post-hoc analysis are indicated in case significant genotype differences were found. * (P < 0.05) ** (P < 0.01) *** (P < 0.001). (TIFF) [file pone.0169051.s006.tiff]

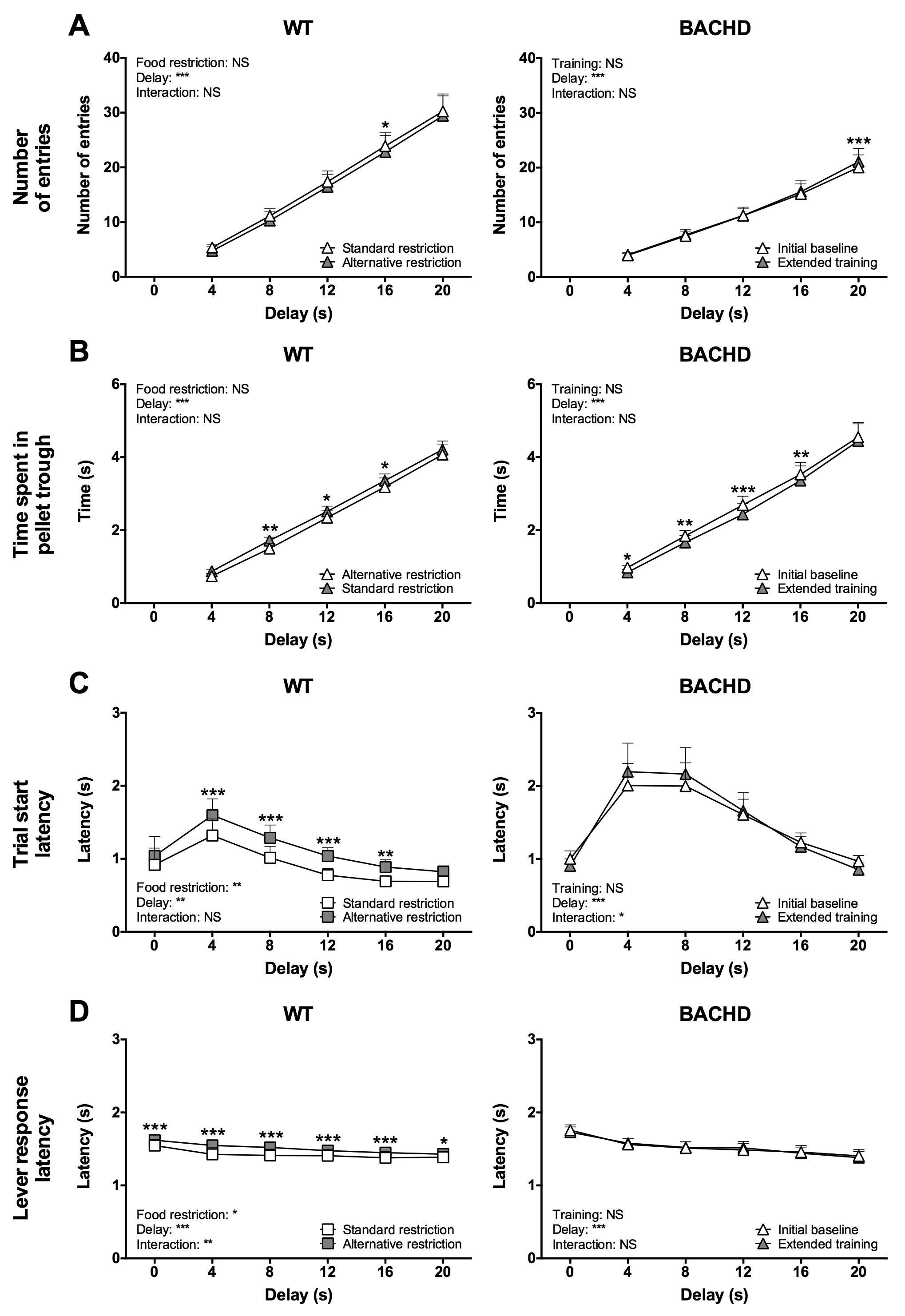

Supplement: S7 Fig — The graphs show some of the parameters of the delayed alternation protocol, comparing performance of WT and BACHD rats during their initial baseline with performance after changing food restriction protocol or given extended training, respectively. All graphs were constructed based on the mean performance over all test ages, as the effect of changing food restriction protocol or giving extended training did not noticeably change with age. Graphs indicate group mean plus standard error. Results from two-way repeated measures ANOVA are shown inside the graphs. Results from post-hoc analysis are indicated in case significant differences between baselines were found. * (P < 0.05) ** (P < 0.01) *** (P < 0.001). (TIFF) [file pone.0169051.s007.tiff]

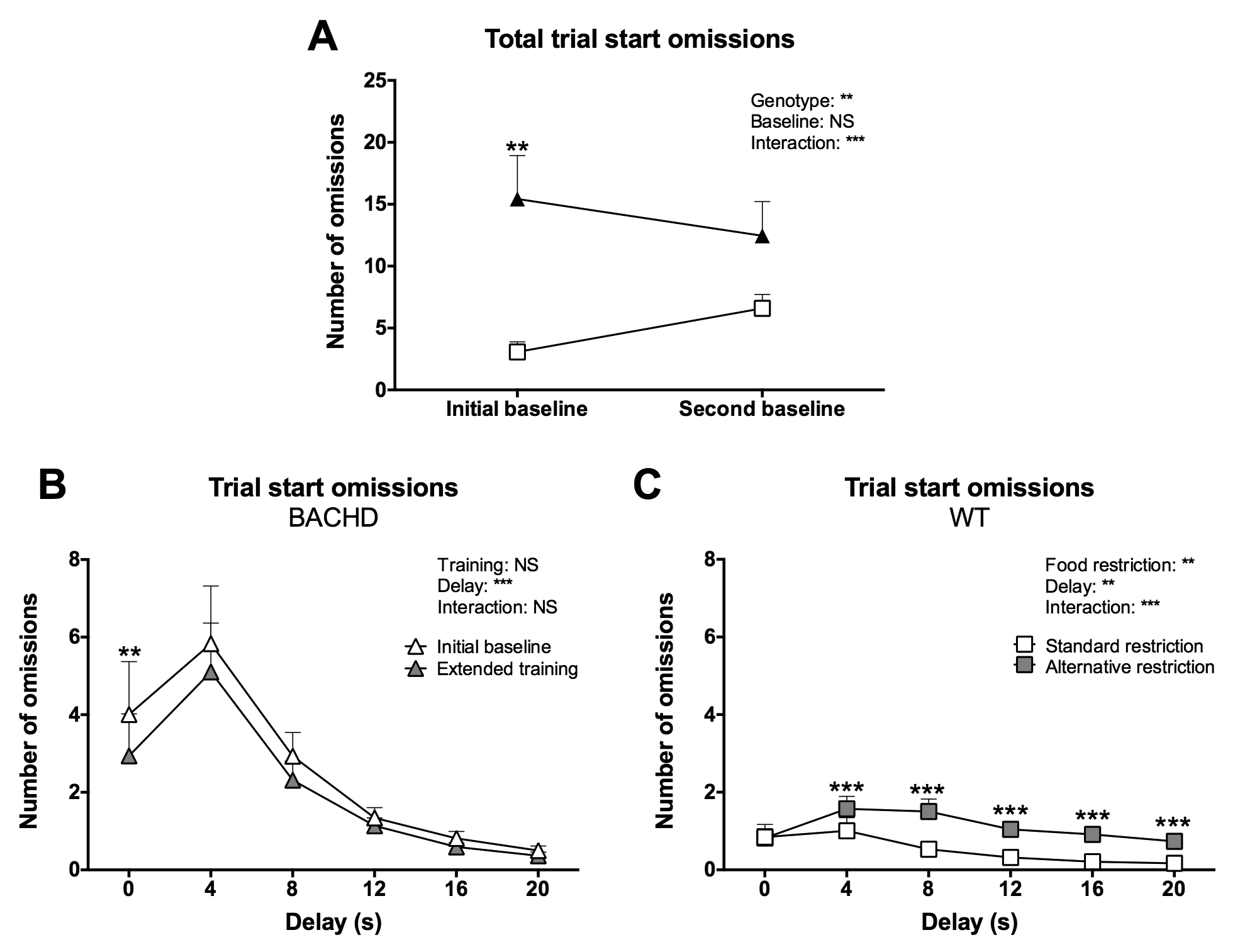

Supplement: S8 Fig — The graphs show the effect of food restriction adjustment and extended training on the number of trial start omissions performed during the delayed alternation test. All graphs were constructed based on the mean performance over all test ages, as the effect of changing food restriction protocol or giving extended training did not noticeably change with age. Graphs indicate group mean plus standard error. Results from two-way repeated measures ANOVA are shown inside the graphs. Results from post-hoc analysis are indicated in case significant differences between baselines were found. * (P < 0.05) ** (P < 0.01) *** (P < 0.001). (TIFF) [file pone.0169051.s008.tiff]

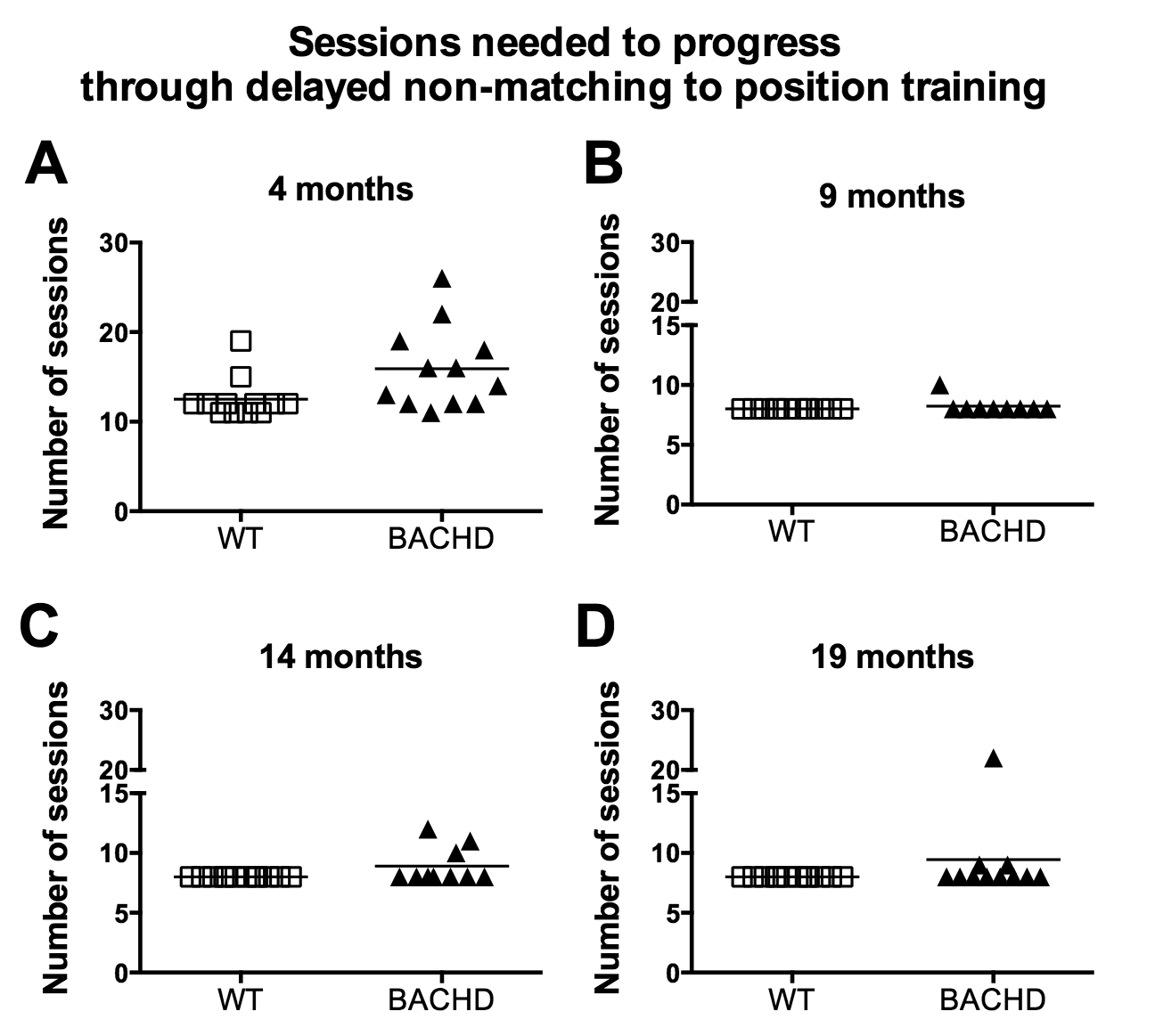

Supplement: S9 Fig — The graphs show the total number of sessions required for progressing through the series of delayed non-matching to position protocols with gradually increasing delay durations, which were implemented before the training on the final delay set had started. The values were adjusted for the change in criterion that was made after the first test age. Rats that did not reach criterion on each protocol were excluded from the analysis. Plots indicate single values for individual rats. Note that the scale on the y-axis differs between (A) and the remaining graphs. Results from t-test or Mann-Whitney U test are indicated in case significant genotype differences were present. * (P < 0.05) ** (P < 0.01) *** (P < 0.001). (TIFF) [file pone.0169051.s009.tiff]

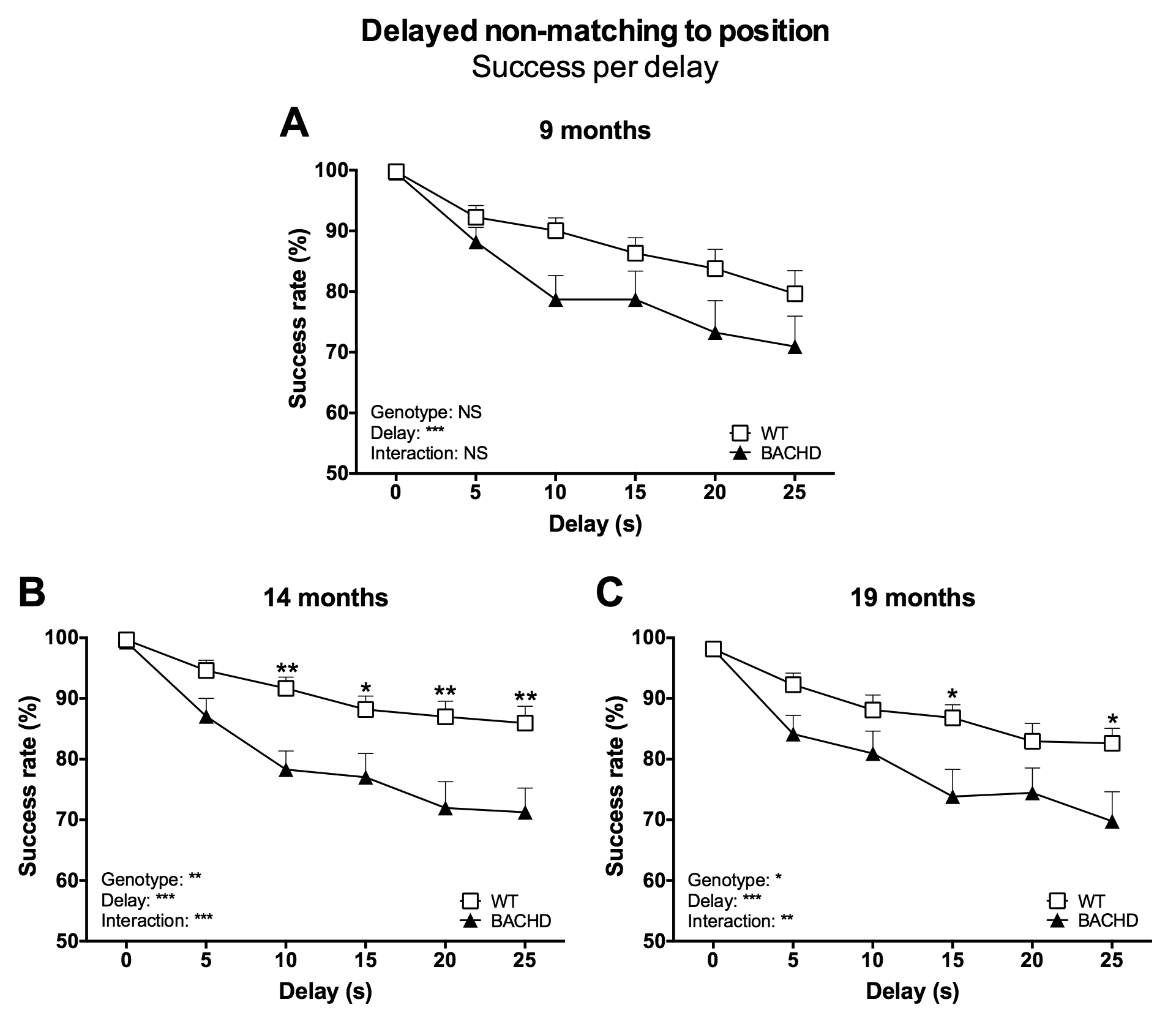

Supplement: S10 Fig — The graphs show the age development of success rate on trial types with delays of different durations in the delayed non-matching test. Each graph shows the stable performance found when rats were maintained on the standard food restriction protocol. Curves display group mean plus standard error. Results from two-way repeated measures ANOVA are shown inside the graphs. Results from post-hoc analysis are indicated in case significant genotype differences were found. * (P < 0.05) ** (P < 0.01) *** (P < 0.001). (TIFF) [file pone.0169051.s010.tiff]

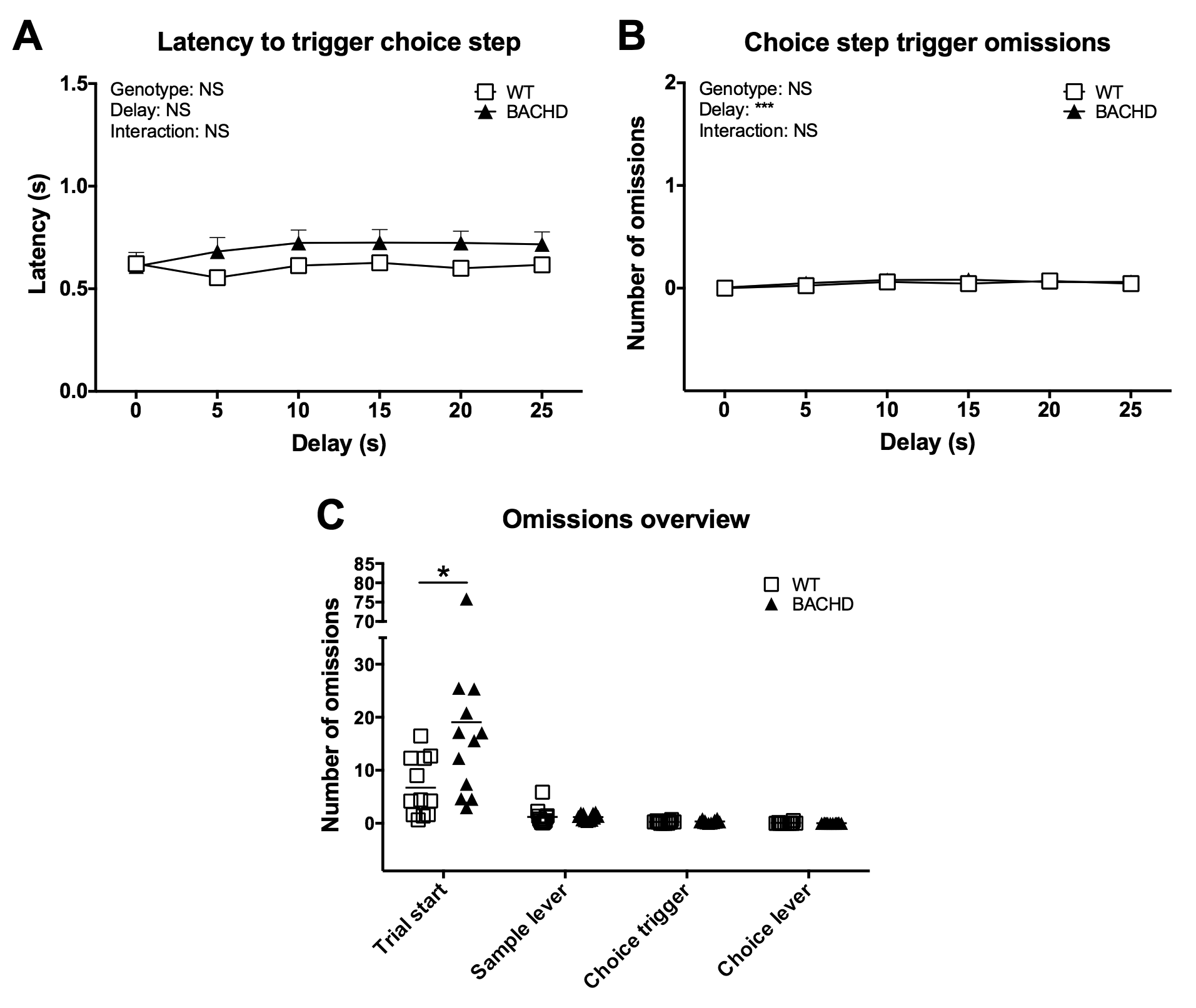

Supplement: S11 Fig — The graphs show the latency to initiate the choice step, related omissions and omissions overview during the delayed non-matching to position protocol. Graphs display the mean performance over all ages, as no significant differences in the rats’ behavior at different ages was found. (A) and (B) indicate group mean plus standard error. (C) indicates the performance of individual rats. For (A) and (B), results from two-way repeated measures ANOVA are shown inside the graphs, and results from post-hoc analysis are indicated in case significant genotype differences were found. For (C), results from t-test or Mann-Whitney U test are indicated in case the genotypes differed significantly. * (P < 0.05) ** (P < 0.01) *** (P < 0.001). (TIFF) [file pone.0169051.s011.tiff]

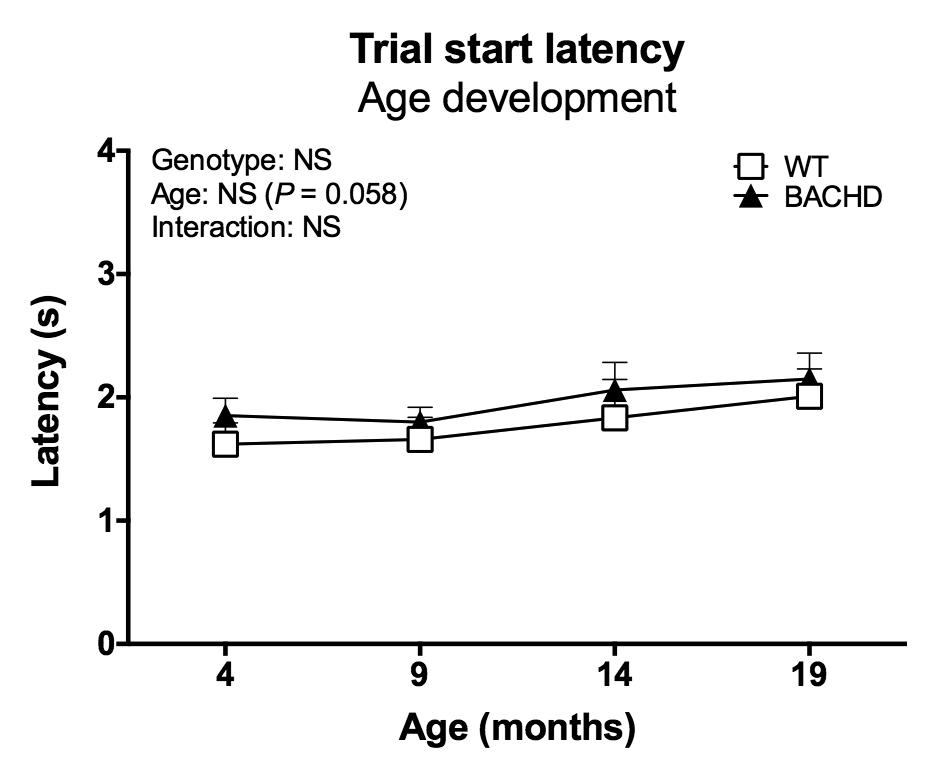

Supplement: S12 Fig — The graph shows the latency to initiate trials on the different test ages of the delayed non-matching to position protocol. The curve indicates group mean plus standard error. Results from two-way repeated measures ANOVA are shown inside the graph, and results from post-hoc analysis are indicated in case significant genotype differences were found. * (P < 0.05) ** (P < 0.01) *** (P < 0.001). (TIFF) [file pone.0169051.s012.tiff]

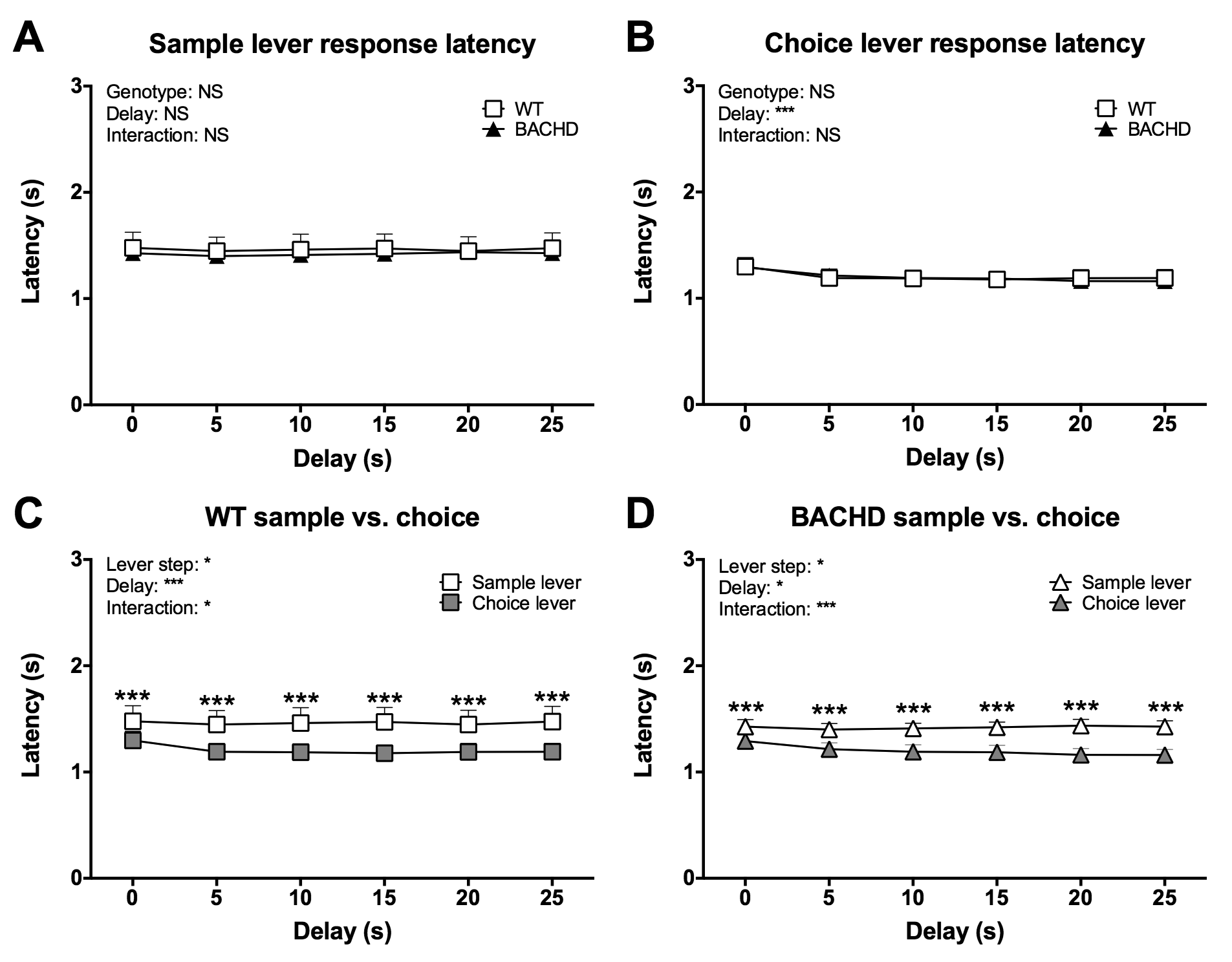

Supplement: S13 Fig — The graphs show the latencies to respond to a lever during either the sample step or the choice step of the delayed non-matching to position protocol. (A) and (B) display the comparison between WT and BACHD for both response latencies, while (C) and (D) display comparisons between the type of response latencies for both genotypes. Graphs display mean performance over all ages, as no significant differences in the rats’ behavior at different ages was found. Curves indicate group mean plus standard error. Results from two-way repeated measures ANOVA are shown inside the graphs, and results from post-hoc analysis are indicated for data points where significant genotype differences were found. * (p < 0.05) ** (p < 0.01) *** (p < 0.001). (TIFF) [file pone.0169051.s013.tiff]

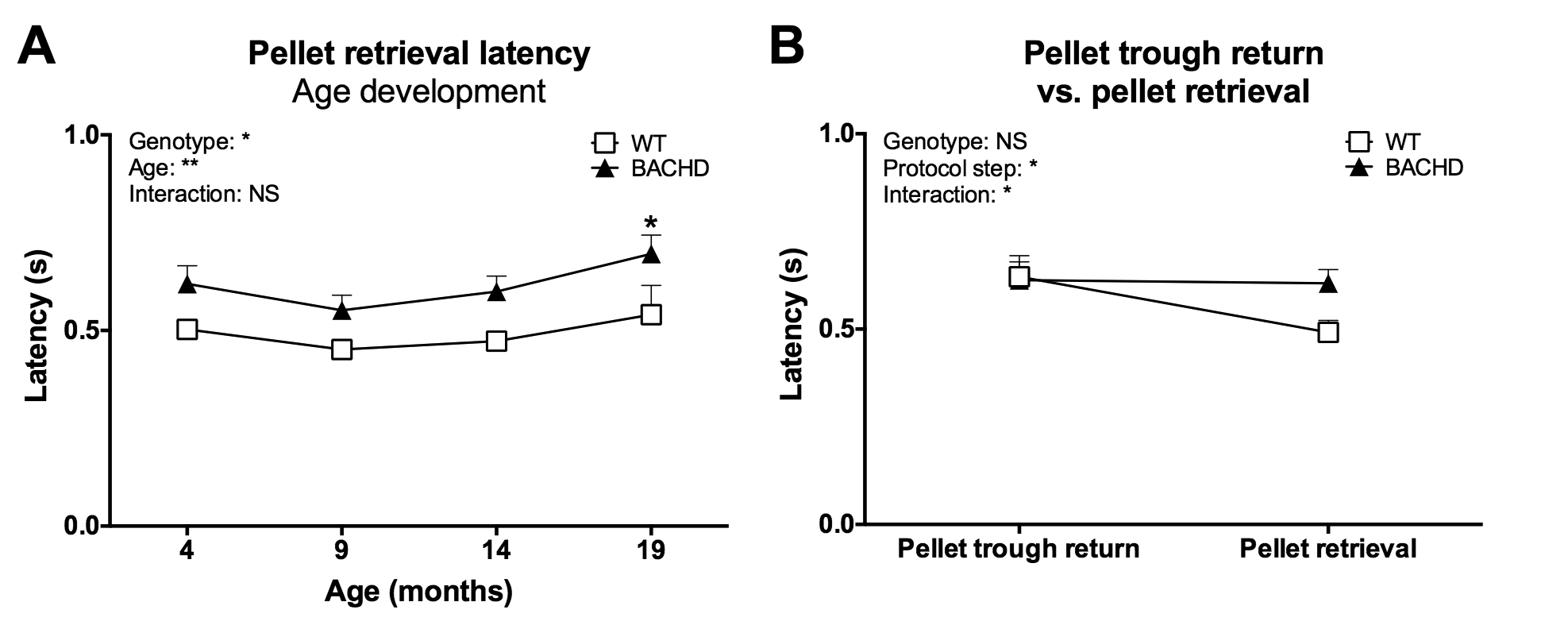

Supplement: S14 Fig — (A) shows the mean pellet retrieval latency of WT and BACHD rats during the delayed non-matching to position protocol at all investigated ages. (B) shows a comparison of the mean pellet retrieval latency with the mean latency to return to the pellet trough after pushing the sample lever. For this, the mean of all investigated ages and trial types were used, as the phenotypes or differences between latencies did not clearly change with age. Curves indicate group mean plus standard error. Results from two-way repeated measures ANOVA are shown inside the graphs, and results from post-hoc analysis are indicated in case significant genotype differences were found. * (P < 0.05) ** (P < 0.01) *** (P < 0.001). (TIFF) [file pone.0169051.s014.tiff]

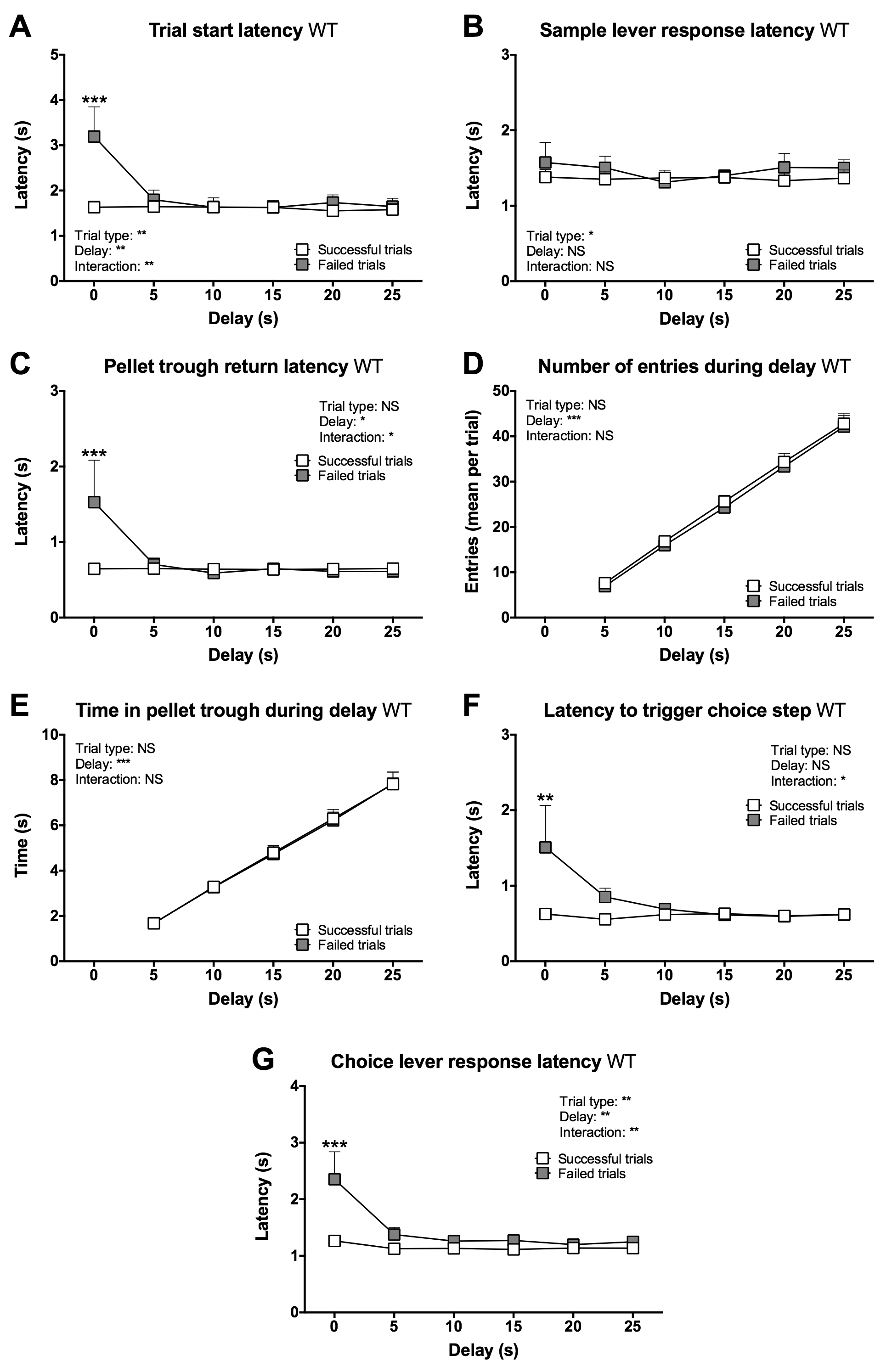

Supplement: S15 Fig — The graphs show some of the parameters of the delayed non-matching to position protocol performance of WT rats separated for successful and failed trials. All graphs were constructed using the mean performance over all ages, as the parameters’ relation to trial outcome did not noticeably change between test ages. In addition, this was necessary to obtain data for failed 0-second delay trials for all rats. Curves indicate group mean plus standard error. Results from two-way repeated measures ANOVA are shown inside the graphs, and results from post-hoc analysis are indicated in case significant genotype differences were found. * (P < 0.05) ** (P < 0.01) *** (P < 0.001). (TIFF) [file pone.0169051.s015.tiff]

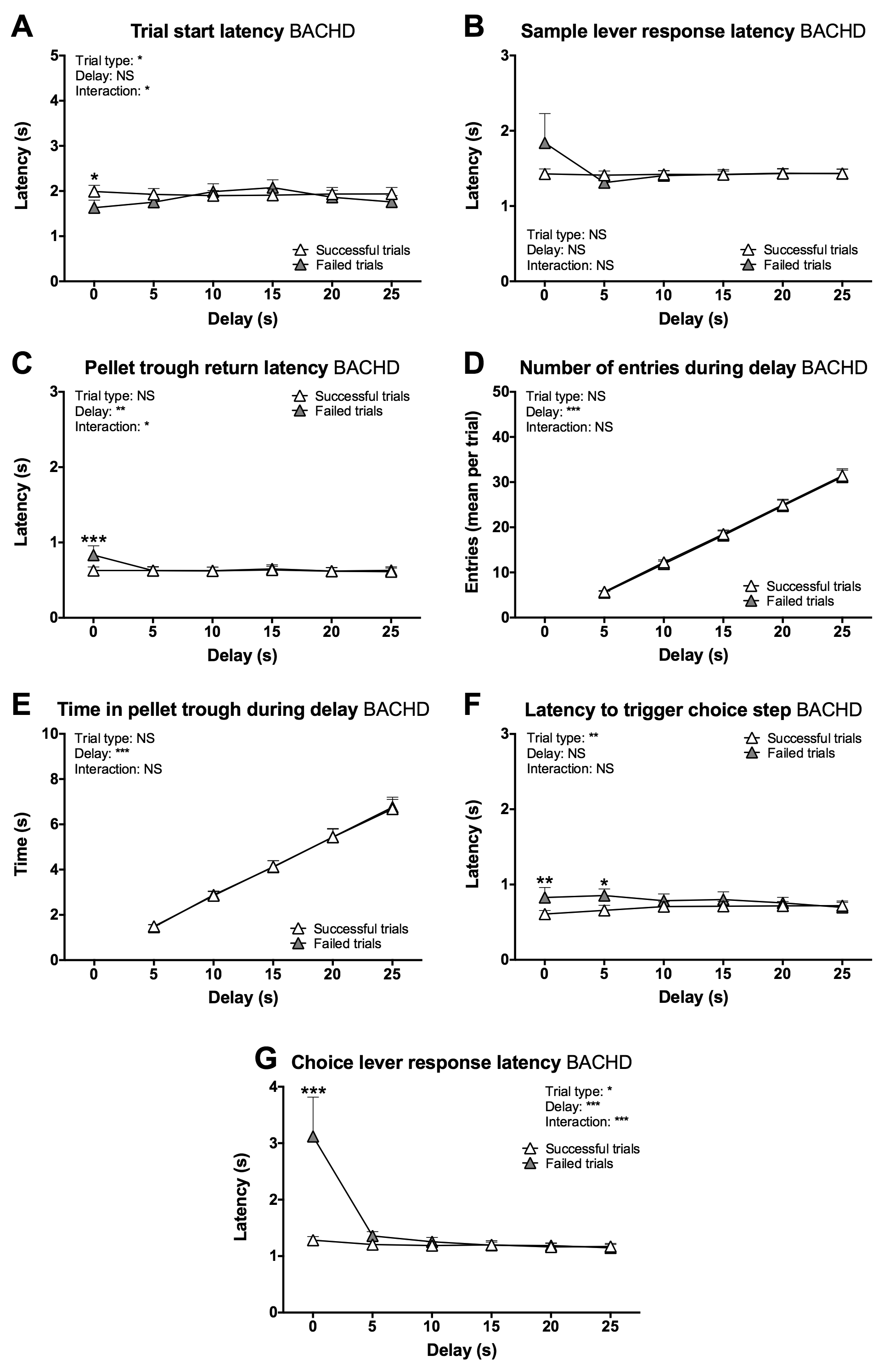

Supplement: S16 Fig — The graphs show some of the parameters of the delayed non-matching to position protocol performance of BACHD rats separated for successful and failed trials. All graphs were constructed using the mean performance over all ages, as the parameters’ relation to trial outcome did not noticeably change between test ages. In addition, this was necessary to obtain data for failed 0-second delay trials for all rats. Curves indicate group mean plus standard error. Results from two-way repeated measures ANOVA are shown inside the graphs, and results from post-hoc analysis are indicated in case significant genotype differences were found. * (P < 0.05) ** (P < 0.01) *** (P < 0.001). (TIFF) [file pone.0169051.s016.tiff]

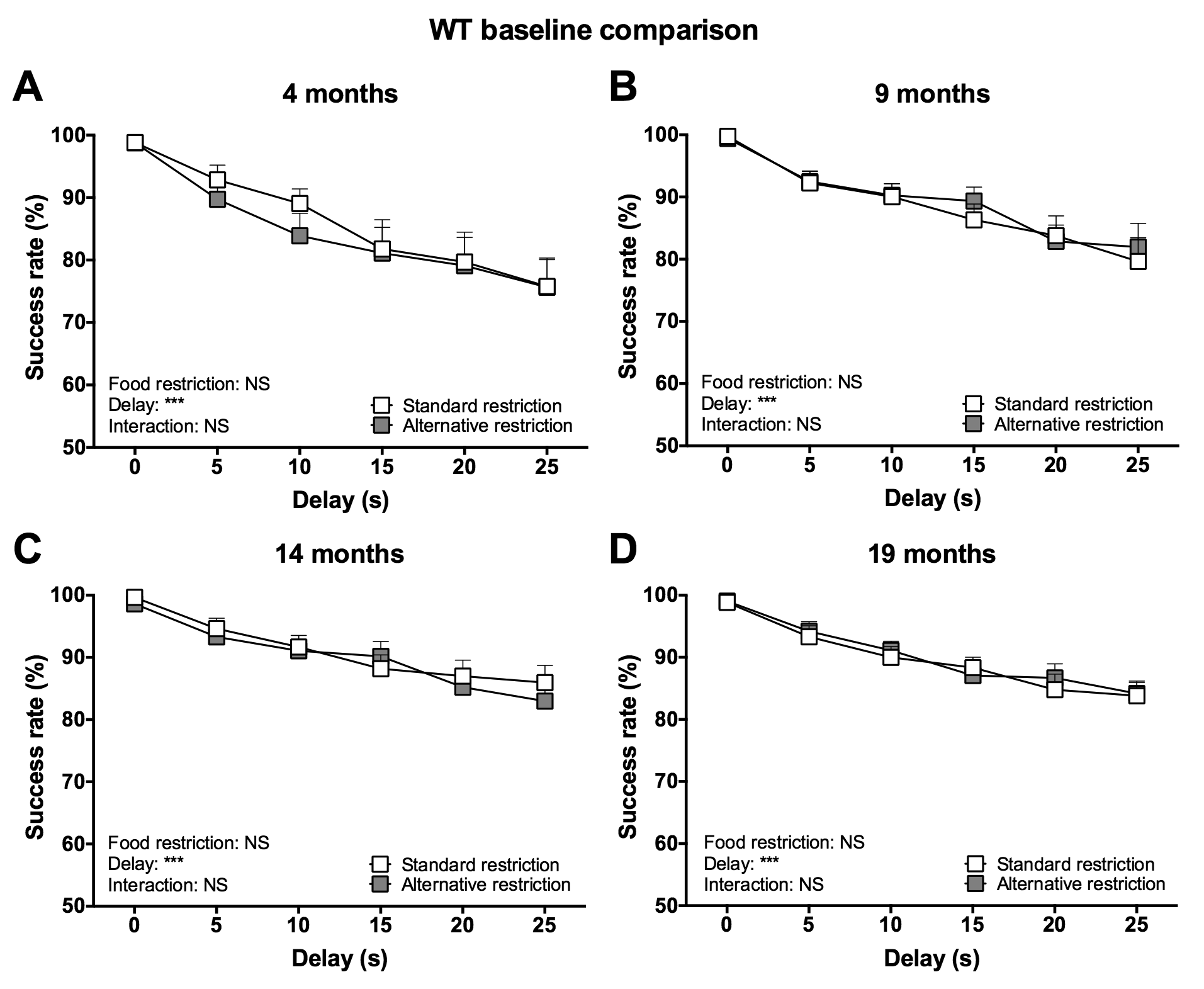

Supplement: S17 Fig — The graphs show the WT rats' performance in the delayed non-matching to position test during two different food restriction settings at the four investigated age. Graphs indicate group mean plus standard error. Results from two-way repeated measures ANOVA are shown inside the graphs. Results from post-hoc analysis are indicated in case significant genotype differences were found. * (P < 0.05) ** (P < 0.01) *** (P < 0.001). (TIFF) [file pone.0169051.s017.tiff]

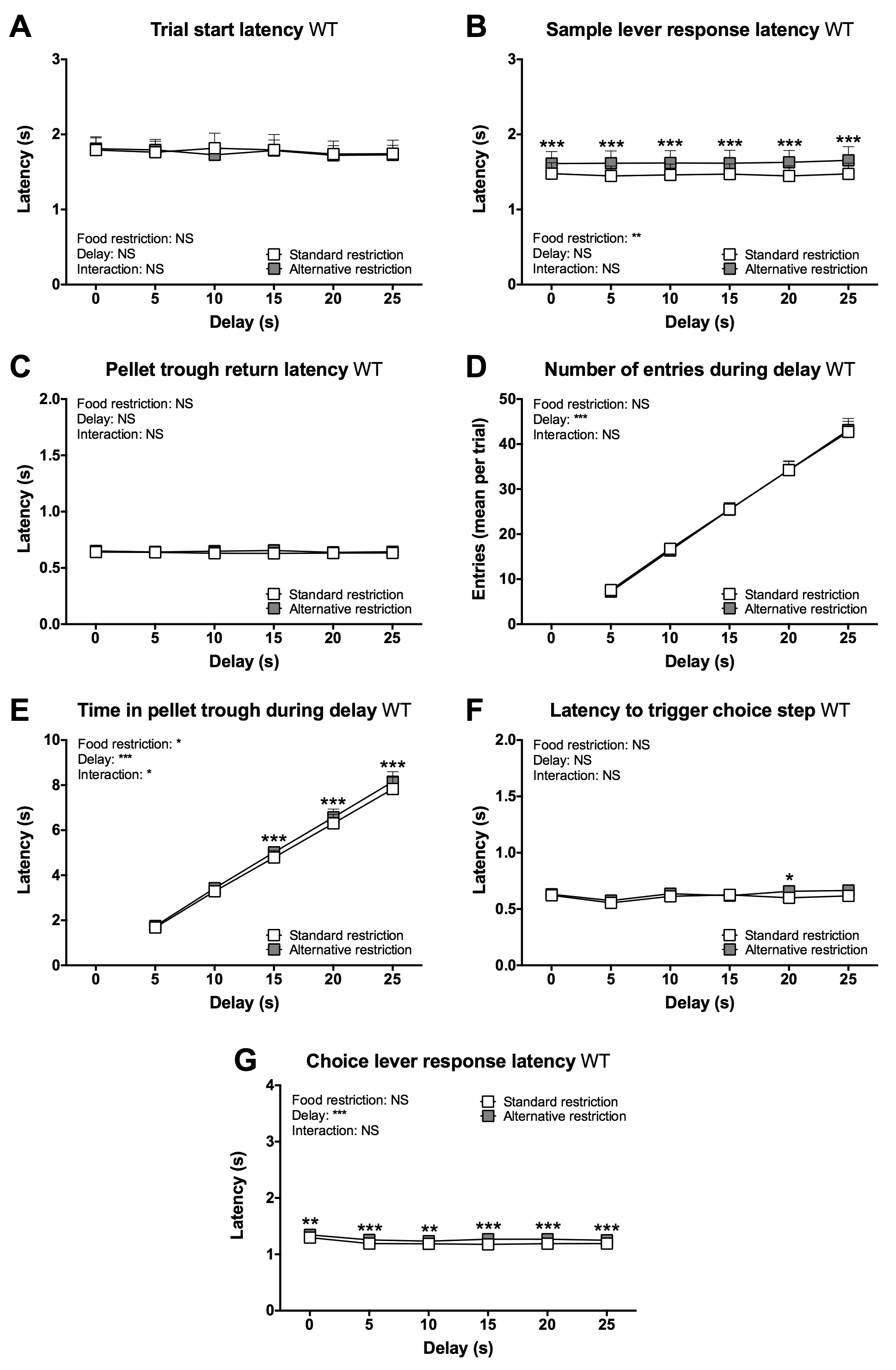

Supplement: S18 Fig — The graphs show some of the parameters of the delayed non-matching to position protocol performance of WT rats separated for standard and alternative food restriction protocols. All graphs were constructed using the mean performance over all ages, as the parameters’ relation to motivational state did not noticeably change between test ages. Curves indicate group mean plus standard error. Results from two-way repeated measures ANOVA are shown inside the graphs, and results from post-hoc analysis are indicated in case significant genotype differences were found. * (P < 0.05) ** (P < 0.01) *** (P < 0.001). (TIFF) [file pone.0169051.s018.tiff]

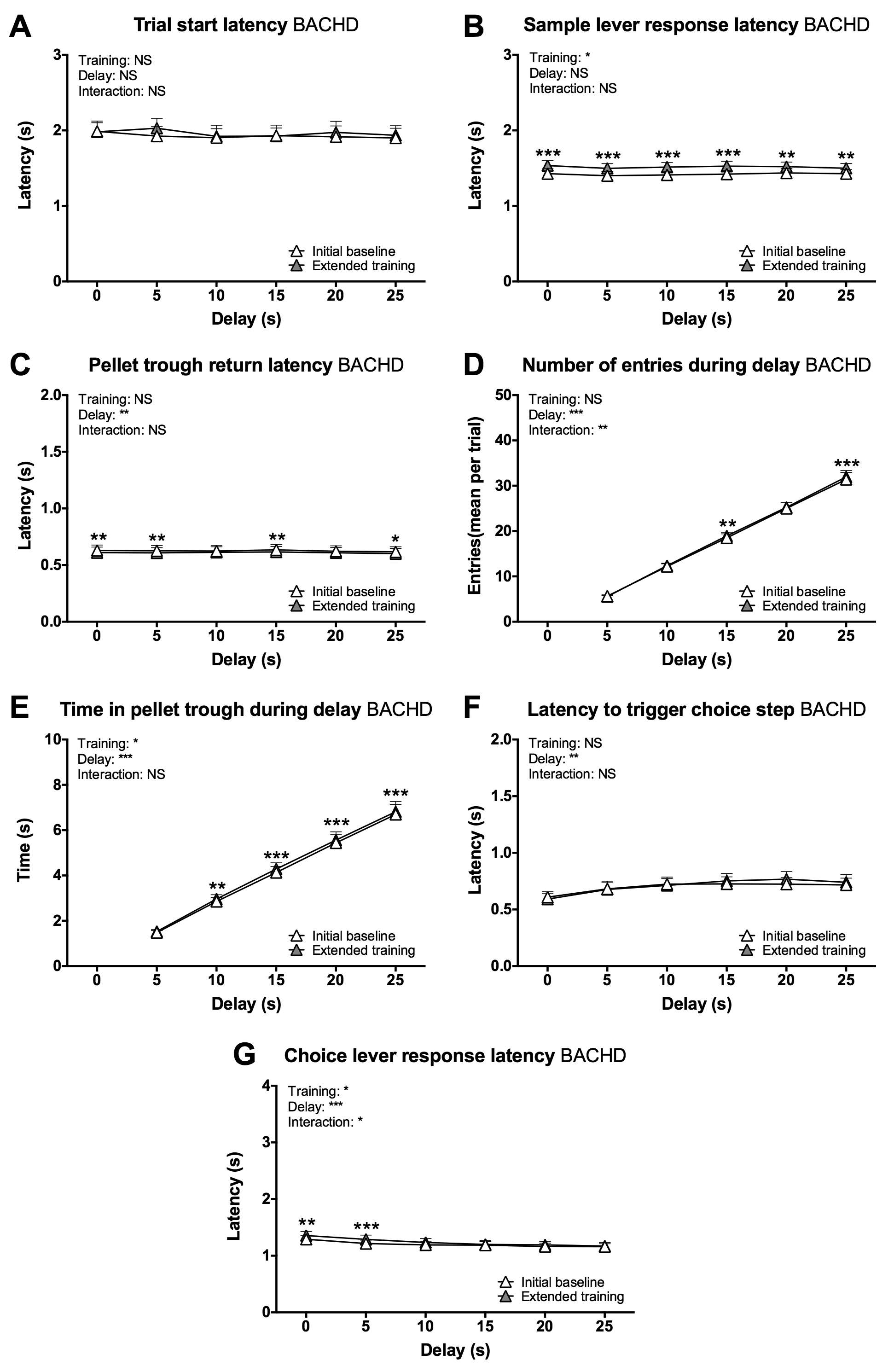

Supplement: S19 Fig — The graphs show some of the parameters of the delayed non-matching to position protocol performance of BACHD rats separated for the baselines after initial and extended training. All graphs were constructed using the mean performance over all ages, as the parameters’ relation to the amount of training did not noticeably change between test ages. Curves indicate group mean plus standard error. Results from two-way repeated measures ANOVA are shown inside the graphs, and results from post-hoc analysis are indicated in case significant genotype differences were found. * (P < 0.05) ** (P < 0.01) *** (P < 0.001). (TIFF) [file pone.0169051.s019.tiff]

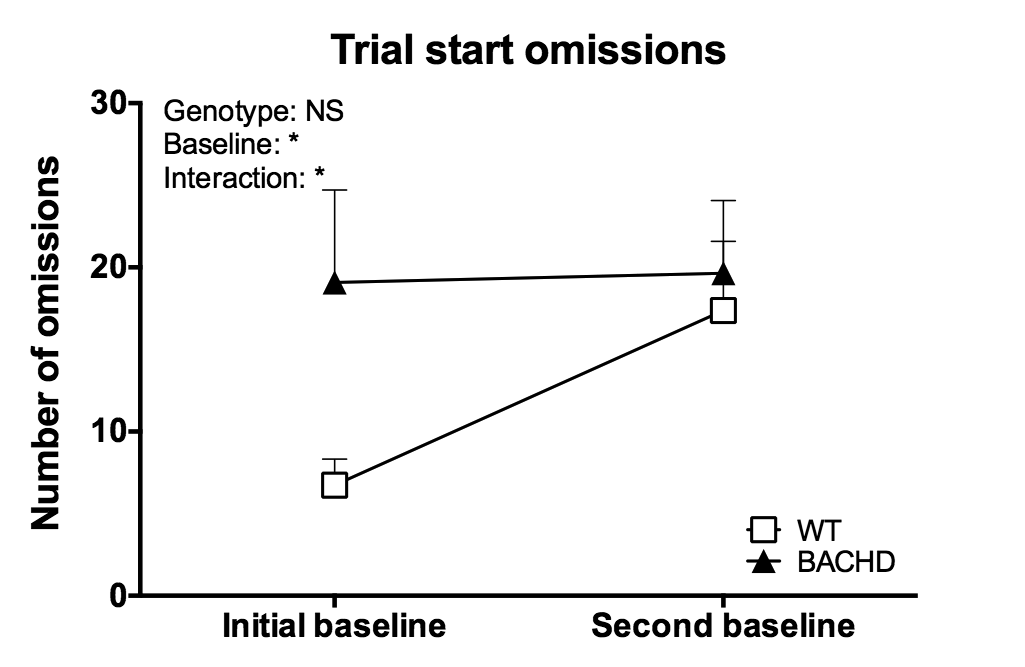

Supplement: S20 Fig — The graph shows the number of omissions during the initial baselines and after either a change in food restriction protocol or extended training on the delayed non-matching to position protocol. The graph was constructed using the mean performance over all ages, as the parameters’ relation to food restriction or extended training did not noticeably change between test ages. The curve indicates group mean plus standard error. Results from two-way repeated measures ANOVA are shown inside the graph, and results from post-hoc analysis are indicated in case significant genotype differences were found. * (P < 0.05) ** (P < 0.01) *** (P < 0.001). (TIFF) [file pone.0169051.s020.tiff]
